# *HLA-G* genetic diversity and evolutive aspects in worldwide populations

**DOI:** 10.1038/s41598-021-02106-4

**Published:** 2021-11-29

**Authors:** Erick C. Castelli, Bibiana S. de Almeida, Yara C. N. Muniz, Nayane S. B. Silva, Marília R. S. Passos, Andreia S. Souza, Abigail E. Page, Mark Dyble, Daniel Smith, Gabriela Aguileta, Jaume Bertranpetit, Andrea B. Migliano, Yeda A. O. Duarte, Marília O. Scliar, Jaqueline Wang, Maria Rita Passos-Bueno, Michel S. Naslavsky, Mayana Zatz, Celso Teixeira Mendes-Junior, Eduardo A. Donadi

**Affiliations:** 1 Molecular Genetics and Bioinformatics Laboratory, Experimental Research Unit, School of Medicine, São Paulo State University (UNESP), Botucatu, State of São Paulo Brazil; 2 Department of Pathology, School of Medicine, São Paulo State University (UNESP), Botucatu, State of São Paulo CEP: 18618970 Brazil; 3 Division of Clinical Immunology, Department of Medicine, Ribeirão Preto Medical School, University of São Paulo (USP), Ribeirão Preto, SP CEP: 14049-900 Brazil; 4 Laboratório Multiusuário de Estudos em Biologia, Centro de Ciências Biológicas, Universidade Federal de Santa Catarina (UFSC), Florianópolis, Brazil; 5 Departamento de Biologia Celular, Embriologia e Genética, Centro de Ciências Biológicas, Universidade Federal de Santa Catarina (UFSC), Florianópolis, Brazil; 6 Department of Population Health, London School of Hygiene and Tropical Medicine, London, UK; 7 Departament of Anthropology, University College London (UCL), London, UK; 8 Bristol Medical School (PHS), University of Bristol, Bristol, UK; 9 Department of Experimental and Health Sciences, Universitat Pompeu Fabra, Barcelona, Spain; 10 Departament of Anthropology, Unversity of Zurich, Zurich, Switzerland; 11 Escola de Enfermagem e Faculdade de Saúde Pública, Universidade de São Paulo (USP), São Paulo, State of São Paulo Brazil; 12 Human Genome and Stem Cell Research Center, Biosciences Institute, University of São Paulo (USP), São Paulo, State of São Paulo Brazil; 13 Department of Genetics and Evolutionary Biology, Biosciences Institute, University of São Paulo (USP), São Paulo, State of São Paulo Brazil; 14 Hospital Israelita Albert Einstein, São Paulo, State of São Paulo Brazil; 15 Departamento de Química, Faculdade de Filosofia, Ciências e Letras de Ribeirão Preto, Universidade de São Paulo, 14040-901 Ribeirão Preto, SP Brazil

**Keywords:** Bioinformatics, Genotype, Haplotypes, Immunogenetics, Population genetics, Immunogenetics

## Abstract

*HLA-G* is a promiscuous immune checkpoint molecule. The *HLA-G* gene presents substantial nucleotide variability in its regulatory regions. However, it encodes a limited number of proteins compared to classical HLA class I genes. We characterized the *HLA-G* genetic variability in 4640 individuals from 88 different population samples across the globe by using a state-of-the-art method to characterize polymorphisms and haplotypes from high-coverage next-generation sequencing data. We also provide insights regarding the *HLA-G* genetic diversity and a resource for future studies evaluating *HLA-G* polymorphisms in different populations and association studies. Despite the great haplotype variability, we demonstrated that: (1) most of the *HLA-G* polymorphisms are in introns and regulatory sequences, and these are the sites with evidence of balancing selection, (2) linkage disequilibrium is high throughout the gene, extending up to *HLA-A,* (3) there are few proteins frequently observed in worldwide populations, with lack of variation in residues associated with major *HLA-G* biological properties (dimer formation, interaction with leukocyte receptors). These observations corroborate the role of *HLA-G* as an immune checkpoint molecule rather than as an antigen-presenting molecule. Understanding *HLA-G* variability across populations is relevant for disease association and functional studies.

## Introduction

The human leukocyte antigen G (*HLA-G*) belongs to the family of non-classical HLA class I molecules, first identified at the maternal–fetal interface, especially on the placenta’s cytotrophoblast cells, where immunotolerance contributes to the fetus maintenance. The *HLA-G* molecule is considered a promiscuous immune checkpoint molecule, inhibiting antigen-presenting cells, T, B, and NK lymphocytes, through the interaction with several leukocyte receptors, including ILT2, ILT4, CD160, KIR2DL4, and CD8 receptors^1^. ILT2 and ILT4 also interact with some classical class I HLA molecules but have more affinity for *HLA-G*^2^, and KIR2DL4 is a specific receptor for *HLA-G*^3,4^. *HLA-G* ligation with these receptors modulates T cytotoxic and NK cell activity, inducing the expression of inhibitory receptors containing Tyrosine motifs^5^.

*HLA-G* deserves special attention in pregnancy because *HLA-G* plays a role in immunosuppression and homeostasis maintenance during pregnancy^6^. Therefore, the non-expression of classical HLA class I molecules by the trophoblastic cells make them potential targets for the lysis mediated by NK cells and the interaction of the *HLA-G* molecule (trophoblast) with uterine NK inhibitor receptors hampers the trophoblast cell lysis^7^. *HLA-G* also activates the production of cytokines that promote remodeling of the vascularization at the maternal–fetal region, of major importance for oxygen supply to the fetus^8–10^. *HLA-G* deregulation is also important in pathological situations such as tumors and autoimmune diseases. *HLA-G* polymorphisms and expression levels have been associated with susceptibility to infections, tumors, and autoimmune diseases^11–16^.

At least seven *HLA-G* isoforms have been described by alternative splicing of the primary transcript, encoding four membrane-bound (G1-G4) and three soluble (G5-G7) isoforms, and their presence in tissue cells may be benefic or pernicious, depending on the underlying condition^15,17^. However, rare isoforms have been detected in tumor samples^18^.

The *HLA-G* coding region presents similarities to the classical HLA class I genes. While most HLA genes are extremely polymorphic, *HLA-G* is highly conserved in its coding nucleotide sequence, presenting a much lower number of different haplotypes, defined mainly by polymorphisms that span around putative regulatory regions and introns. In the IPD-IMGT/HLA database^19^, release 3.45.1 from July 2021, *HLA-G* exhibited 88 alleles (different sequences), encoding 26 allotypes, but few are frequent, as demonstrated later in this study. The *HLA-G* coding region is relatively conserved, exhibiting few functional polymorphisms (e.g., non-synonymous variants); however, the *HLA-G* regulatory regions are more variable, displaying target sites for many transcriptional and post-transcriptional regulatory elements^20^. Thus, some polymorphic sites at these segments have been associated with different *HLA-G* expression profiles.

While the *HLA-G* gene regulatory region variability may impact the magnitude of gene/protein expression, the coding region variability may impact the *HLA-G* biological properties. Differential transcriptional and post-transcriptional *HLA-G* activities have been associated with nucleotide variability at the promoter and 3’ untranslated regions^21–24^. Additional sites potentially involved in *HLA-G* expression have also been reported for the *HLA-G* distal-promoter (located between − 2635 and − 1406 positions) and the proximal-promoter (located between − 1406 and − 1)^25^. Several regulatory elements can induce *HLA-G* expression, including progesterone responsive elements (PRE), heat shock element (HRE), interferon-sensitive response element (ISRE), while others may repress protein expression (RREB1—Ras-responsive element-binding protein 1; GLI3—Glioma Associated Oncogene Homologue 3)^17^. Besides the distal and proximal promoters, a 121-bp region 12 Kb upstream the *HLA-G* gene (Enhancer L) is reported to be a target for transcription factors (CEBP and GATA) associated with the placentation process^26^.

A previous initiative addressing *HLA-G* diversity in worldwide samples was reported^27^; however, this survey used a different sequencing technology and distinct genotyping and haplotyping methods; i.e., it was based on low-coverage data from the 1000 Genomes consortium phase I, which included a much lower number of samples^27^. Other studies addressed specific samples from Brazil, Cyprus, and Benin, and characterized the full gene sequence and the proximal promoter but disregarded the distal promoter region^28–30^. Many studies addressed *HLA-G* polymorphisms using Sanger sequencing in association studies, usually evaluating only a segment of the gene or specific exons (e.g.^31–35^, and many others).

This study explored the *HLA-G* genetic diversity and the evolutionary aspects in worldwide population samples by using a state-of-the-art method to characterize polymorphisms and haplotypes from high-coverage next-generation sequencing (NGS) data. We also provide the most comprehensive survey of the *HLA-G* genetic diversity across worldwide populations and resources for future analyses evaluating *HLA-G* polymorphisms in terms of disease association and functional studies.

## Samples and methods used to characterize *HLA-G* genetic diversity

We evaluated 4738 individuals from 88 different population samples from four datasets, all of them sequenced in high-coverage (> 30×) and processed through the same computational pipeline. The first dataset was the new high-coverage sequencing data of the 1000 Genomes consortium^36^, the second consisted of 831 samples from the International Genome Sample Resource (IGSR) consortium^37^, the third encompassed 1323 elderly Brazilians from the *Saúde, Bem-estar e Envelhecimento* (SABE) cohort^38^, and the fourth comprised the high coverage sequencing of 80 individuals from Southeast Asia and Oceania, including the Agta hunter-gatherer people from the Northern Luzon island, Philippines. The population samples and their sample sizes are in Table S1. In all cases, we started from a genome-wide BAM file with reads aligned to the reference hg38 genome (version hg38DH) by using BWA MEM (https://sourceforge.net/projects/bio-bwa/).

The method used to extract the *HLA-G* data is described in detail in a file (the supplementary methods) with a description of all methods and variables we used. This method also follows the same strategy used to characterize HLA class I genes in Brazilian samples^38^. Using a single pipeline to extract the data from all samples is essential to avoid biases in the genotyping and haplotyping procedures. In brief, we used the hla-mapper version 4.0 to optimize read alignment in the HLA complex, minimizing alignment errors and cross-alignments that are commonly observed among HLA genes^39^. We have called genotypes using GATK HaplotypeCaller in the GVCF mode (https://gatk.broadinstitute.org) with a further refinement step using vcfx (www.castelli-lab.net/apps/vcfx). This method allows the detection of any previously unknown *HLA-G* variant. The haplotype inference used both read-aware phasing (GATK ReadBackedPhasing) and probabilistic models with the workflow phasex/shapeit4 (www.castelli-lab.net/apps/phasex). Please refer to the file (supplementary methods) for details regarding the mapping, genotyping, and haplotyping procedures. Applying the bioinformatic pipeline, 4640 samples (97.9%) passed the quality control and the haplotyping procedure, as presented in Table S1.

To support the quality of the data presented here, we evaluated departures of the Hardy–Weinberg expectations (HWE) at the SNP level in population samples with at least 10 individuals (63 populations out of 88). Of those, 55 (87.3%) present no HWE deviation, and 8 populations present just two deviations. Thus, most SNPs fit HWE.

### Resources used to evaluate the HLA diversity

The method described above allowed us to retrieve SNP and haplotype data from approximately 7870 nucleotides surrounding the *HLA-G* gene, from 4328 bases upstream of the first translated ATG to 100 nucleotides downstream *HLA-G*. This large region includes most of the regulatory elements, all exons and all introns. We extracted coding (exons + introns), exonic (the CDS), promoter, and 3’UTR sequences. We also translated CDS sequences into proteins (the allotypes).

In the following sections, we will discuss the *HLA-G* polymorphisms across the world, providing insights regarding the *HLA-G* genetic diversity, and present a resource for future studies evaluating *HLA-G* polymorphisms and disease association studies in different populations. We also provide several useful tools to evaluate HLA-G diversity (Table 1) that can be downloaded from the website www.castelli-lab.net/HLA-G or are included as supplementary material.

**Table 1:** Resources available for download or listed as supplementary material regarding the *HLA-G* gene and its polymorphisms.

Resource	Description	Availability
VCF listing all variants	A VCF file containing all variants, the reference and alternative alleles, and global counts, to be used as support for variant refinement when genotyping *HLA-G* in NGS procedures or variant annotation	For download^a^
Phased VCF	A VCF file with the phased genotypes for each sample	Upon request
FASTA file with full sequences	A copy of every full-length sequence observed in this survey (approximately 12 Kb each), and their global counts	For download^a^
FASTA file with gene sequences	The full sequence of each *HLA-G* allele (exons + introns) observed in this survey, with their global counts. Each sequence is identified with its official name according to the IPD-IMGT/HLA database, or as a novel sequence	For download^a^
FASTA file with CDS sequences	We extracted all different exonic sequences (CDS). This file contains a copy of each different sequence, with their global counts. Each sequence is identified with its official name according to the IPD-IMGT/HLA database, or as a novel sequence	For download^a^
FASTA file with protein sequences	We translated all exonic sequences into proteins (allotypes). This file contains a copy of each different sequence, with their global counts. Each sequence is identified with its official name according to the IPD-IMGT/HLA database, or as a novel sequence	For download^a^
FASTA file with 3’UTR sequences	The sequence of each 3’UTR haplotype we have detected, with their names and global counts	For download^a^
SNP frequencies	This table provides the frequency for the reference and alternative alleles in the global population, in XLSX format	Supplementary material (Table S2)
Genomic alleles (4-digit resolution) frequencies	This table provides the frequency for each genomic allele (4-digit resolution) in each population we have studied, in XLSX format. There are 3 sheets, one for biogeographic regions, one for countries, and one for specific population samples	Supplementary material (Table S3)
Allotypes frequencies (2-digit resolution)	This table provides the frequency for each allotype (2-digit resolution, full-length protein) in each population we have studied, in XLSX format. There are 3 sheets, one for biogeographic regions, one for countries, and one for specific population samples	Supplementary material (Table S4)
3’UTR frequencies	This table provides the frequency for 3’UTR haplotype in each population we have studied, in XLSX format. There are 4 sheets, one for biogeographic regions, one for countries, one for specific population samples, and one describing the combination between genomic alleles and 3’UTR haplotypes in the global sample	Supplementary material (Table S5)

^a^Available for download in www.castelli-lab.net/HLA-G.

## Nomenclature, SNPs, nucleotide diversity, and linkage disequilibrium across *HLA-G*

Due to the lack of a standardized nomenclature for the regulatory regions, individual regulatory region haplotypes have been previously designated as “distal/proximal promoter regions”, and “3’UTR haplotypes”, as proposed by our group^29,40,41^. Because the *HLA-G* region evaluated in this study includes an *extensae* region (from − 4328 upstream the first translated ATG to 100 nucleotides downstream the transcription end site), and there is no designated nomenclature to nominate the sequences we have characterized (for instance, when the sequence includes the complete promoter, all introns, and the complete 3’UTR sequence), we used the term GEMBIO_HLA-G_H1 (complete haplotype number 1, for HLA-G, available in the GeMBio laboratory Database) to designate this large gene sequence, which is available for download (Table 1). These names can be converted to whatever future nomenclature will be available and standardized. Whenever this complete nomenclature is used in this text, the previous names of the regulatory (Distal-G, PROMO-G, and 3’UTR) and coding region (as defined by the IPD-IMGT/HLA database) haplotypes are also mentioned.

We detected 442 different variants encompassing approximately 7870 nucleotides surrounding the *HLA-G locus*. The majority are bi-allelic single nucleotide exchanges, but there are some indels, multi-allelic variants and combinations of them. The list of each variant, with its reference and alternative alleles, and their global frequencies, is available in Table S2. The VCF containing the phased genotypes of every individual is available upon request.

**Figure 1:**
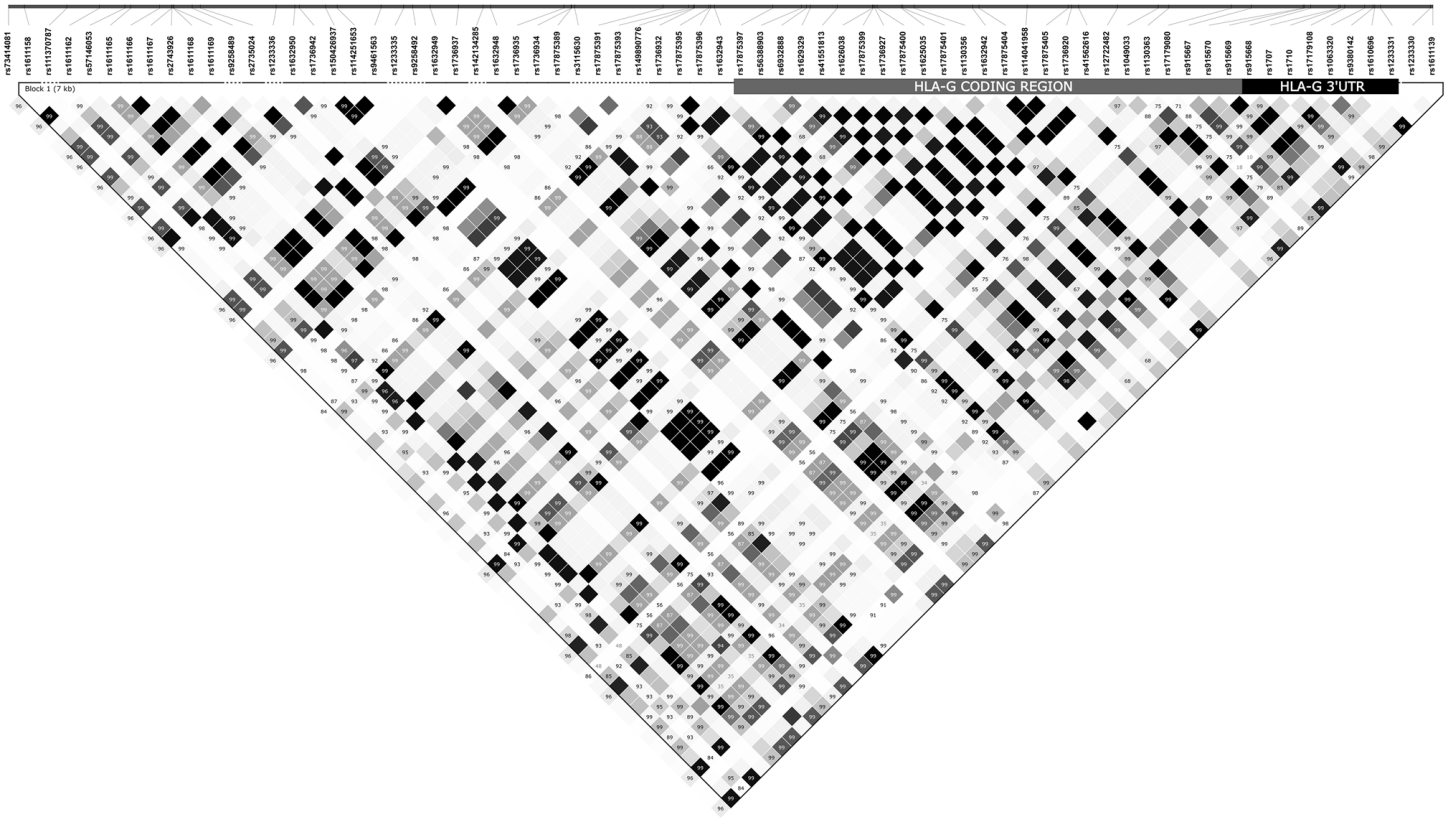
Linkage disequilibrium between pairs of 106 bi-allelic SNPs at the *HLA-G* gene region, starting from 4 Kb upstream the gene up to 100 nucleotides downstream, all presenting a global minimum allele frequency of 1%. The image was generated in the Haploview and further edited by using Inkscape. Areas in black indicate strong LD (*r*^2^ > 0.8), shades of gray indicate moderate LD, and white indicates low LD.

These 442 variants are arranged into 514 different haplotypes or full-length sequences (Table 1) and some of these haplotypes are quite common. The two most frequent ones are: (1) GEMBIO_HLA-G_H1 presents a frequency of 21.6% and is associated with known haplotypes, such as the distal promoter Distal-010101a, the proximal promoter PROMO-G010101a, the coding allele G*01:01:01:01, and the 3’UTR haplotype known as UTR-01, and (2) GEMBIO_HLA-G_H2 presents a frequency of 10.8% and carries the distal promoter Distal-010102a, the proximal promoter PROMO-G010102a, the coding allele G*01:01:02:01, and UTR-02^29,30^. We observed 20 full-length haplotypes presenting global frequencies higher than 1%, exhibiting a summed frequency of 76.85% (from GEMBIO_HLA-G_1 to GEMBIO_HLA-G_20). Thus, despite the presence of many variants, there are few full-length frequent haplotypes, reflecting the high Linkage Disequilibrium (LD) along the *HLA-G locus*. In Fig. 1, we present the LD pattern along the *HLA-G locus* for bi-allelic variants with a minor allele frequency (MAF) > 0.1% and fitting HWE (106 variants).

The entire *HLA-G* gene is included within a single segregation block. Although Fig. 1 illustrates the global LD pattern, we observed similar patterns when considering only samples from Europe, Africa, Asia, and American. Most of the pairwise comparisons present *D’* = 1 (Fig. 1 and S1), and many comparisons present *r*^*2*^ > 0.8. We demonstrate that this high LD extends at least 4 kb upstream *HLA-G* to 100 nucleotides downstream. Moreover, there are many variants in complete LD (*r*^*2*^ = 1.00). Of the 106 variants plotted in Fig. 1, 43 (40.6%) present another variant in complete LD. For instance, rs17875397 (position − 56) is in absolute LD with nine other variants, including rs17875400 (+ 507) and rs17875405 (+ 1534). There are also many variants in almost complete LD (measured by *r*^*2*^ > 0.95). For instance, considering the threshold *r*^*2*^ > 0.95, 55 selected SNPs can tag all 106 frequent variants along *HLA-G*. Because of that, LD among *HLA-G* variants must be considered when performing any association study to disregard hitchhiking effects. This high LD explains the presence of a few haplotypes that are frequent globally when analyzing almost 8 Kb surrounding *HLA-G.* This high LD was previously proposed evaluating other population samples^29,40^, and it may extend at least more 20 Kb downstream the gene, where an Alu insertion accompanies the G*01:01:01:01 allele^42^, or even extend up the *HLA-A locus*^43^.

**Figure 2:**
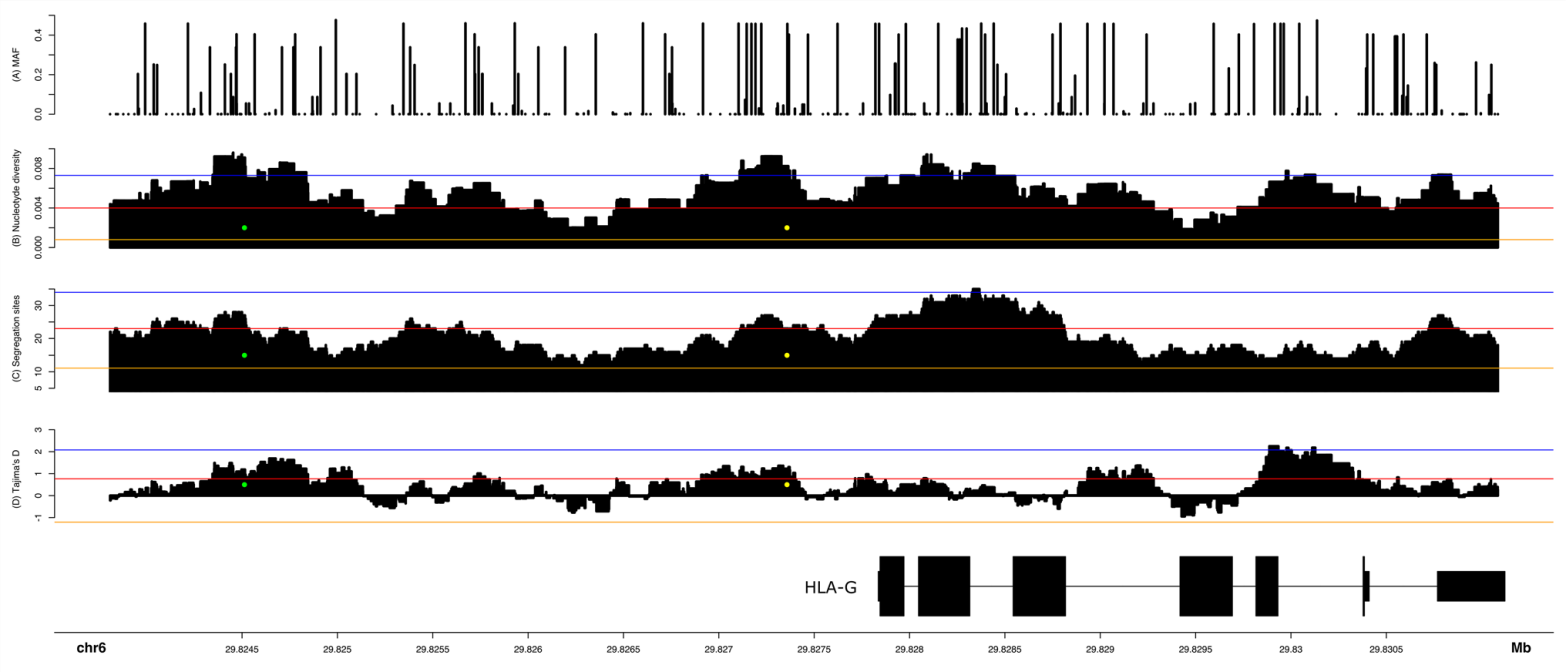
Frequency of each variant (panel A), nucleotide diversity (panel B), number of segregation sites (panel C), and Tajima’s *D* (panel D) across *HLA-G,* considering all samples from the 1000Genomes project pooled together, starting from approximately 4 kb upstream the gene (the promoter region) to 100 nucleotides downstream *HLA-G.* Panels B, C, and D were computed in sliding windows of 500 nucleotides and a step size of 1. The *HLA-G* exon/intron structure is indicated in the bottom panel, with fine lines indicating introns and thick lines indicating exons. To evaluate the significance of the parameters estimated from *HLA-G*, we built a null distribution considering the patterns observed in chromosome 6, computing these statistics in 10,000 random windows of 500 nucleotides from chromosome 6. The values above the blue and red horizontal lines are higher than 99.9% and 99% of the ones observed for chromosome 6, respectively. The orange line represents the average observed in chromosome 6 for each statistic. The green and the yellow dots represent polymorphisms -3614 (rs1611163) and − 725 (rs1233334), respectively.

Figure 2 illustrates the frequency of each *HLA-G* variant (panel A), nucleotide diversity (panel B), SNP density (Panel C), and Tajima’s *D* (panel D) across the *HLA-G* region, in windows of 500 nucleotides and step size of 1, starting from 4 kb upstream *HLA-G* (the promoter region) to 200 nucleotides downstream it, considering all 1000Genomes samples pooled together. To evaluate the significance of the parameters estimated from *HLA-G*, we built a null distribution considering the patterns observed in chromosome 6. For that purpose, we computed these statistics in 10,000 random windows of 500 nucleotides from chromosome 6. The values above the blue and red horizontal lines are higher than 99.9% and 99% of the ones observed in the null distribution for chromosome 6, respectively. The orange line represents the average observed for each statistic. Similar patterns can be observed in all biogeographic regions. This analysis has revealed interesting insights. Nucleotide diversity across *HLA-G* is usually higher than 0.4% (the red line), which is much higher than the average observed from chromosome 6 (0.082%). The regions with the highest nucleotide diversity (around 1%), which are among the 0.1% highest observed in chromosome 6 (blue line), coincides with the promoter region around positions − 3614 (green dot, rs1611163) and − 725 (yellow dot, rs1233334). Another very polymorphic region coincides with intron 2. Likewise, the strongest positive Tajima’s *D* signals in the promoter region, which is compatible with balancing selection, coincide with these two markers. The region surrounding marker − 3614 (rs1611163) presents many frequent variants, including rs1611164, rs1611165, rs1611166, rs1611167, rs2743926, rs1611168, rs11169, in a short segment of 336 bp. Likewise, the region surrounding maker − 725 presents many frequent variants, including rs2249863 (− 716), rs2735022 (− 689), rs35674592 (− 666), rs17875391 (− 646), and rs1632944 (− 633), all previously described elsewhere^40^. The position − 725 is a tri-allelic variant, which has been described as influencing *HLA-G* expression levels possibly by epigenetic mechanisms^44^. Moreover, except for rs17875391, all these polymorphisms are listed as eQTLs for *HLA-G* expression by the GTEX portal (https://gtexportal.org). It is not clear whether these variants directly influence *HLA-G* expression or if they are in LD with other regulatory elements, such as enhancer L, located 12 Kb upstream *HLA-G*^26^. However, the strongest signal of balancing selection coincides with intron 5 (Fig. 2, panel D), with the Tajima’s *D* values higher than 99.9% of the ones observed for chromosome 6.

When we consider the transcribed *HLA-G* segment, illustrated in the bottom panel at Fig. 2, and the positions and frequencies of variants across *HLA-G* (panel A), we noticed that most frequent variants coincide with intronic regions and that exon 4 is quite conserved. Table 2 presents the number of segregation sites, nucleotide diversity, and Tajima’s *D* in each *HLA-G* exon and intron. Introns 1 and 2 present two of the highest nucleotide diversity indexes across *HLA-G* (Table 2, Fig. 2) and the highest number of segregation sites in the sliding window analysis (Fig. 2). Some of the intronic SNPs are also considered eQTLs for *HLA-G* expression, according to GTEX data (https://gtexportal.org). It is not clear whether these intronic variants directly influence *HLA-G* expression. However, as addressed throughout the following sections, these intronic variants (as haplotypes) are in almost complete LD with some promoter and 3’UTR haplotypes.

**Table 2:**
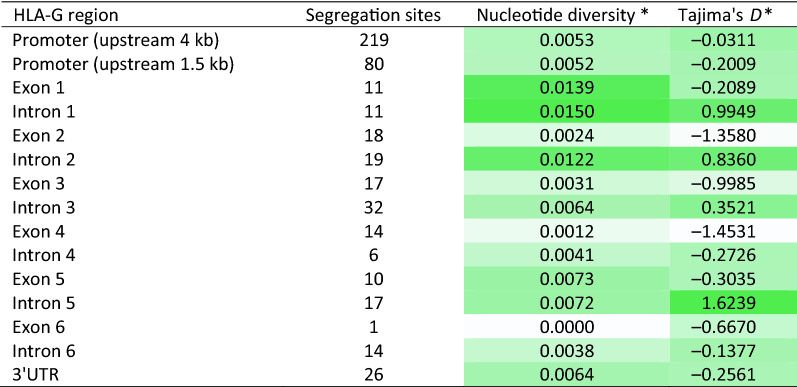
The number of segregation sites, nucleotide diversity, and Tajima’s *D* across different *HLA-G* regions in 4640 samples from 88 different populations.

*The indexes are scaled in shades of green, from white (the lowest value) to dark green (the highest value).

On the other hand, the nucleotide diversity drops to low levels in exons, except for exon 1, which is very short (Table 2) and presents two frequent synonymous variable sites. Additionally, the highest Tajima’ *D* coincides with introns, mostly intron 1, intron 2, and intron 5, all presenting positive Tajima’s *D*, which is compatible with balancing selection. In contrast, all exons present negative values, which is compatible with purifying selection (Table 2). Exon segments are conserved, and most of the exonic variants are synonymous mutations. Exon 2 presents only two frequent variants, one non-synonymous associated with the allotype G*01:03, and the other a synonymous mutation. Exon 3 presents four common variants, two synonymous mutations, one non-synonymous associated with allotype G*01:04, and one frameshift associated with allotype G*01:05. Exon 4 presents only three frequent variants, and one configures a non-synonymous mutation associated with allotype G*01:06. Other rare variants in exons are usually synonymous mutations.

We also calculated the ratio of substitution rates at non-synonymous and synonymous sites in exons (Table 3). Interestingly, none of the exons, when isolated, presented evidence of purifying selection. However, when pooled together, there is evidence of purifying selection at exons 2, 3, and 4 (Table 3). This phenomenon was observed in a previous study that evaluated a reduced number of sequences^45^. Taken together, the nucleotide diversity and Tajima’s *D* patterns (Fig. 2 and Table 2), and the ratio of substitutions (Table 3), made it clear that *HLA-G* is indeed conserved in exons, although not necessarily conserved in introns and regulatory sequences. Nevertheless, nucleotide diversity for introns and the regulatory sequences are much higher than the human average (0.075%)^46^.

**Table 3:** Synonymous and nonsynonymous nucleotide substitution test of neutrality, positive, and purifying selection for analysis over 77 *HLA-G* sequences defined by exon mutations, in 4640 samples from 88 different populations.

*d*_*N*_*/d*_*S*_ neutrality test	Number of sequences	Number of codons	H_A_ = neutrality (*d*_*N*_ ≠ *d*_*S*_)*	H_A_ = positive selection (*d*_*N*_ > *d*_*S*_)	H_A_ = purifying selection (*d*_*N*_ < *d*_*S*_)*
Exon 1	11	24	− 0.8576, P = 0.3928	− 0.9059, P = 1.0000	0.8804, P = 0.1902
Exon 2	19	90	− 1.1546, P = 0.2505	− 1.1160, P = 1.0000	1.1258, P = 0.1312
Exon 3	20	92	− 0.6806, P = 0.4973	− 0.6930, P = 1.0000	0.6790, P = 0.2491
Exon 4	17	92	− 1.0288, P = 0.3056	− 1.007, P = 1.0000	0.9609, P = 0.1692
Exon 5	10	39	− 1.5091, P = 0.1338	− 1.3480, P = 1.0000	1.4232, P = 0.0786
All exons	77	339	− **2.5808, P = 0.0110**	− 2.5069, P = 1.0000	**2.6134, P = 0.0050**
Exons 2 and 3	39	182	− 1.3036, P = 0.1948	− 1.2308, P = 1.0000	1.2635, P = 0.1044
Exons 2, 3, and 4	55	274	− 1.7453, P = 0.0834	− 1.7482, P = 1.0000	**1.6929, P = 0.0465**

*Significant *P*-values are marked in boldface.

**Figure 3:**
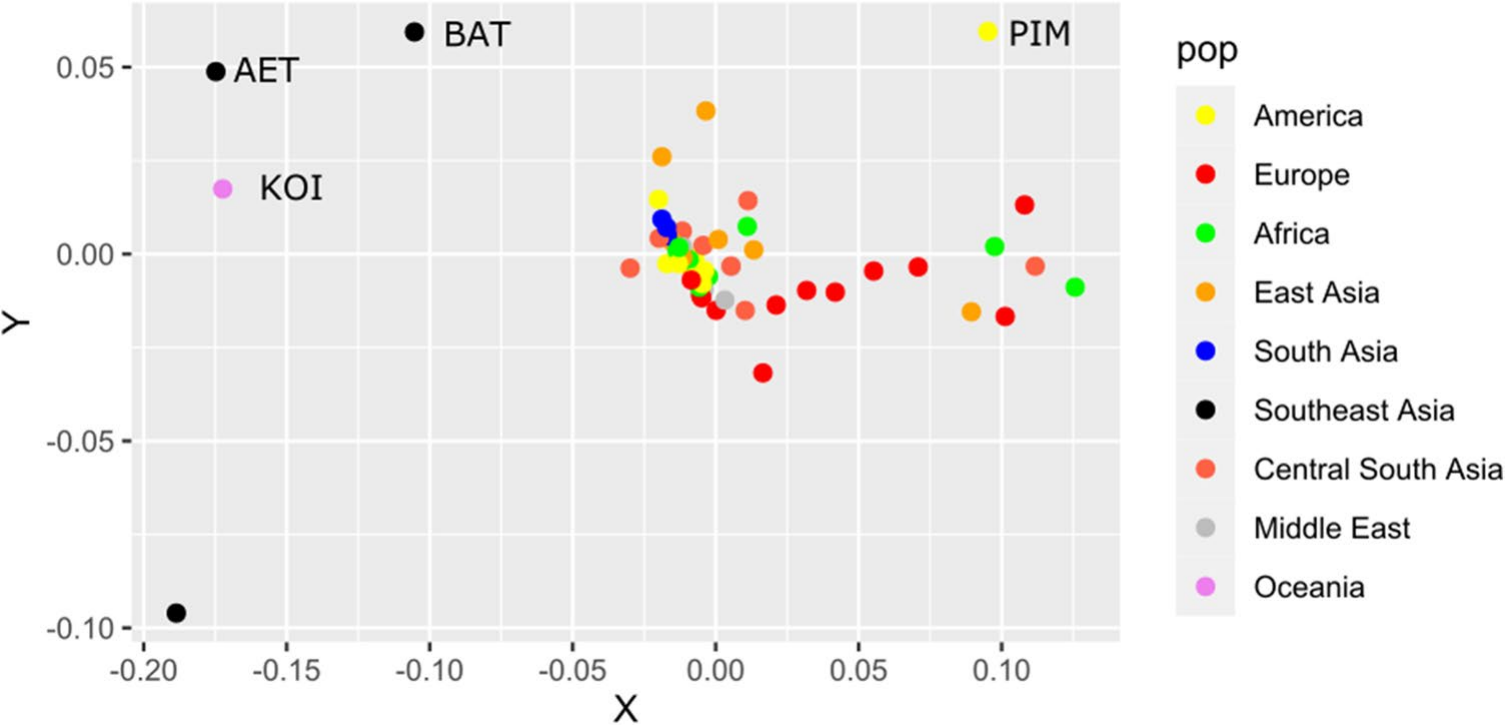
Multidimensional scaling (MDS) illustrating the distance (measured by *F*_*ST*_ estimated from *HLA-G* SNPs) among population samples with at least 10 individuals. The name of each population is available at Table S1. We have indicated the names of some outliers.

Most of the *HLA-G* common SNPs are shared worldwide. Because of that, there is a low population differentiation (Fig. 3), except for very isolated populations that may have experienced strong bottleneck effects, such as the Aeta, Batak, and Pima.

### The *HLA-G* coding region diversity

We considered the diversity observed between position −300 and + 838, which is the region tracked by the IPD-IMGT/HLA database^19^, and included, in this survey, 191 variation sites. All coding sequences we have observed, together with their names and global counts, are available for download (Table 1). We detected 190 different *HLA-G* alleles; 36 of them were already reported (fully or partially) in the IPD-IMGT/HLA database^19^, with a summed frequency of 95.54%. The 154 remaining sequences configure possible new *HLA-G* alleles. Because these new sequences have not been cloned and submitted to the IPD-IMGT/HLA, we named all new sequences as GEMBIO_HLA-G_G followed by a number that identifies this sequence in the GeMBio laboratory. Interestingly, at least 41 of these new alleles occurred more than once, and some are very frequent, such as GEMBIO_HLA-G_G144, with 51 copies detected in Africa, America, and the Middle East. We also confirmed the existence of many rare *HLA-G* alleles that have been submitted to the IPD-IMGT/HLA database, such as G*01:01:01:14Q (4 copies), G*01:09 (1 copy), G*01:11 (11 copies), G*01:14 (6 copies), G*01:21 N (2 copies), G*01:26 (2 copies), and others (Table S3).

**Figure 4:**
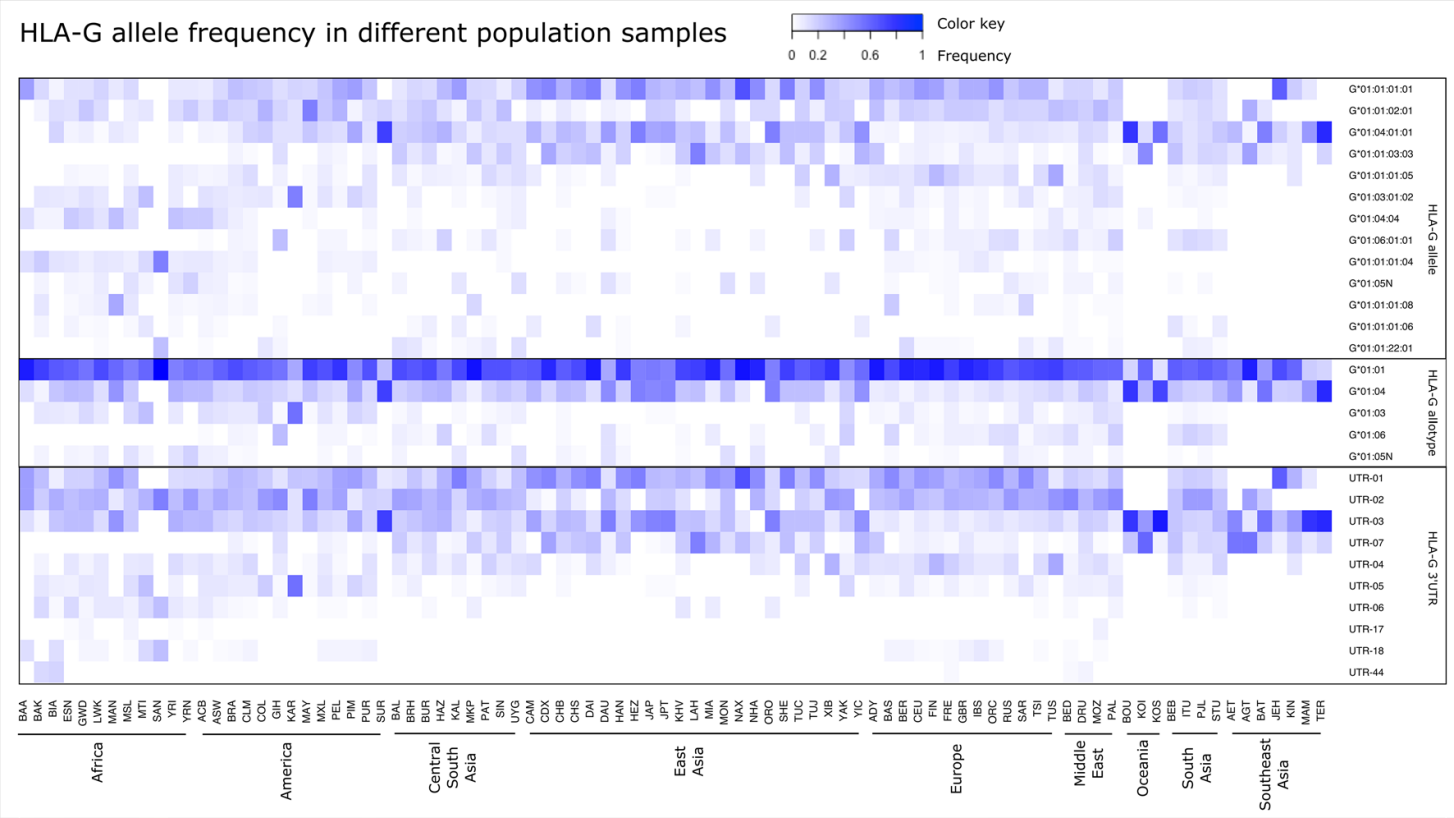
Frequencies of the most common *HLA-G* alleles, allotypes, and 3’UTR haplotypes in different population samples across the world. Tables S3, S4, and S5 present all frequency values.

**Figure 5:**
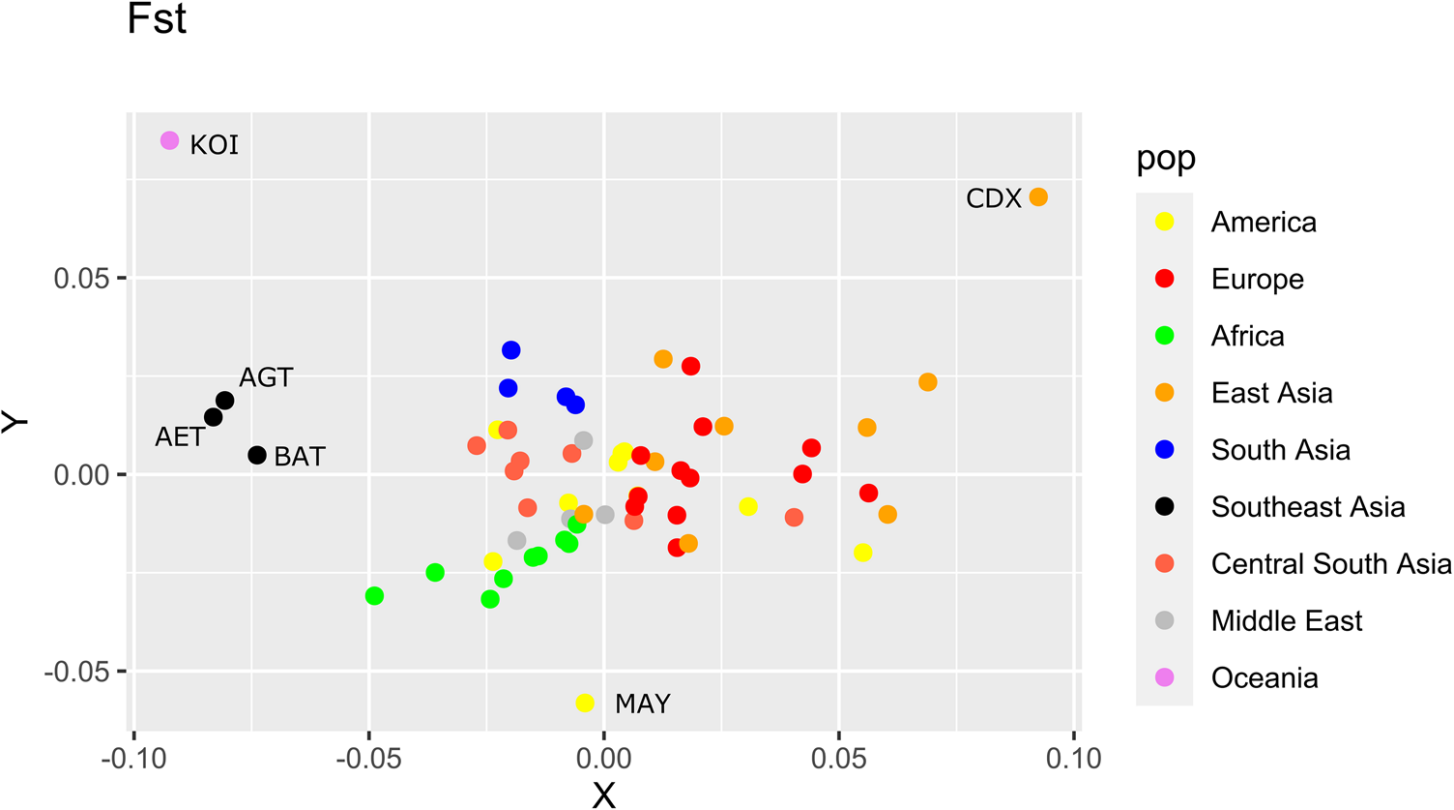
Multidimensional scaling (MDS) illustrating the distance (measured by *F*_*ST*_ estimated from *HLA-G* alleles—coding haplotypes) in populations samples with at least 10 individuals. The name of each population is available at Table S1. We have indicated the names of some outliers.

In terms of global diversity, there are 14 *HLA-G* alleles with global frequencies higher than 1%: G*01:01:01:01, G*01:01:01:04, G*01:01:01:05, G*01:01:01:06, G*01:01:01:08, G*01:01:02:01, G*01:01:03:03, and G*01:01:22:01 (all of them encoding the G*01:01 allotype), G*01:03:01:02 (encoding the G*01:03 allotype), G*01:04:01:01, G*01:04:01:02, and G*01:04:04 (encoding the G*01:04 allotype), G*01:05 N (the null allele), and G*01:06:01:01 (encoding the G*01:06 allotype). These alleles represent 91% of all *HLA-G* sequences detected worldwide, and their frequencies differ in each biogeographic region (Fig. 4), and in each population sample (Table S3). For instance, the frequency of G*01:01:01:01 is high in East Asia, low in Africa, and rare in Oceania, while G*01:01:01:04 is quite rare in Asia and frequent in Africa (Fig. 4). The differentiation among populations, evaluated by *F*_*ST*_*,* became clearer when evaluated using the haplotypes/genomic alleles (Fig. 5) compared to SNPs (Fig. 3), with African, European, South Asian, and Southeast Asian population samples clustering together. However, most of the population samples amalgamate in the center of the plot, because the common *HLA-G* alleles are present worldwide (Fig. 5).

There are 74 alleles that have occurred at least twice. For instance, the new genomic allele named GEMBIO_HLA-G_G128 presents a frequency of 30% among the Aeta and around 10% in Agta and the Bouganville Island samples (Table S3). The hunter-gatherers in the Philippines (Agta) have been linked to Bouganville and Highland Papua New Guinea and Australian hunter-gatherers, possibly associated with a previous out of Africa^47^. The sequence GEMBIO_HLA-G_G31 occurred in many countries, achieving a frequency of 16.7% in the Central African Republic (Table S3). Both these new alleles encode the allotype G*01:01, which is the most frequent worldwide (Fig. 4). As explained earlier, these names represent sequences stored in the GeMBio laboratory Database (www.castelli-lab.net/HLA-G). Thus, *HLA-G* genetic diversity is higher than we currently acknowledge, although only a few different allotypes can be found worldwide. This observation reinforces the conservation in terms of protein diversity, but not necessarily in terms of DNA diversity. In fact, as demonstrated in the previous section, *HLA-G* nucleotide diversity is much higher than the one observed across chromosome 6, particularly in intronic regions. In addition, there is evidence of purifying selection acting on *HLA-G* exons, reinforcing the evidence of protein conservation in worldwide populations.

We have characterized the pair of full sequences of every individual using the cutting-edge sequencing technology, and we have described here, for the first time, the complete sequences (DNA and protein) of some alleles that are only partially reported in the IPD-IMGT/HLA Database. Additionally, we have provided the sequences of many new *HLA-G* alleles. Sequence characterization was simplified because many individuals presented the same full-length sequence for alleles G*01:01:14, G*01:01:15, G*01:01:19, G*01:04:05, G*01:11, and G*01:14, which are only partially characterized in the IPD-IMGT/HLA Database. Likewise, some new sequences occurred in more than ten individuals. This worldwide data clearly reveals that the IPD-IMGT/HLA is outdated in terms of stored data for *HLA-G*. It should be emphasized that, as new NGS initiatives grow, invaluable high-quality and large-scaled HLA data will be shortly produced. Thus, it is of utmost importance that the IPD-IMGT/HLA considers these data to update the official database.

### The *HLA-G* allotype/protein diversity

We have detected 53 different full-length proteins (allotypes), 13 of them already reported by the IPD-IMGT/HLA database (Table S4). As already observed in many studies (www.allelefrequencies.net), only five allotypes are globally frequent (G*01:01, G*01:03, G*01:04, G*01:05 N, and G*01:06), with a summed frequency of 98.9%.

Some allotypes are particularly frequent in specific population samples (Table S4, Fig. 4). G*01:01 is the most frequent in all regions, except Oceania. G*01:06, for instance, is the product of a nucleotide exchange + 1799 C > T at codon 258 in exon 4, exchanging Threonine/Methionine (rs12722482, Table S1), and is frequent in the Middle East and South Asia, with intermediate frequencies in Europe and America, and almost absent in East Asia and Africa. Likewise, this allele was frequent in Cyprus^30^, absent in other Amerindian tribes from Brazil^45^ and Benin^28^, and very rare in other Amerindians from South America^48^. G*01:06 was previously associated with a risk for allograft rejection^49^ and pregnancy complications^31,50,51^, albeit the mechanisms underlying these associations are unknown. G*01:03, which results from an A > T exchange at position + 292 at exon 2, in codon 31, exchanging a Threonine for Serine (rs41551813, Table S1), is common among Africans, Americans (including Brazil), and in the Middle East, but rare among other biogeographic regions. Conversely, it is the most frequent allele among Karitiana (Table S4) and quite frequent in other Amerindian tribes from the Brazilian Amazon^45^. The frequency of G*01:04, which is a consequence of the nucleotide exchange + 755 C > A at exon 3, exchanging Leucine/Isoleucine (rs12722477, Table S1), is particularly high in Asia and Oceania, especially among Japanese. It is also high in Africa, Agta, and many Amerindians from South America^28,45,48^ and less frequent in Europe. G*01:05 N, a truncated HLA-G protein caused by a deletion at exon 3 leading to a premature stop codon further in intron 4 (rs41557518, Table S1), is rare in most regions but frequent in Africa. European populations present the highest frequency of allotype G*01:01, and usually the lowest frequency of other allotypes. Because of that, Europeans (especially Finns) present the lowest observed heterozygosity for allotypes across the world (data not shown).

Some HLA-G allotypes only occurred in specific populations. For example, G*01:11 occurred among Caribbeans, African Americans, Puerto Ricans, and Brazilians, and G*01:14 among Caribbeans, Sierra Leoneans, and African Americans. Moreover, we detected at least 40 new allotypes. These new allotypes are usually rare and restricted to single populations. However, some have occurred more than once, such as the new allotype here named GEMBIO_HLA-G_P5 (P for protein sequence), which occurred in many samples from South East Asia and Oceania (Table S4), and it is a truncated protein sequence. The sequence of each allotype is available as a resource for download (Table 1).

**Figure 6:**
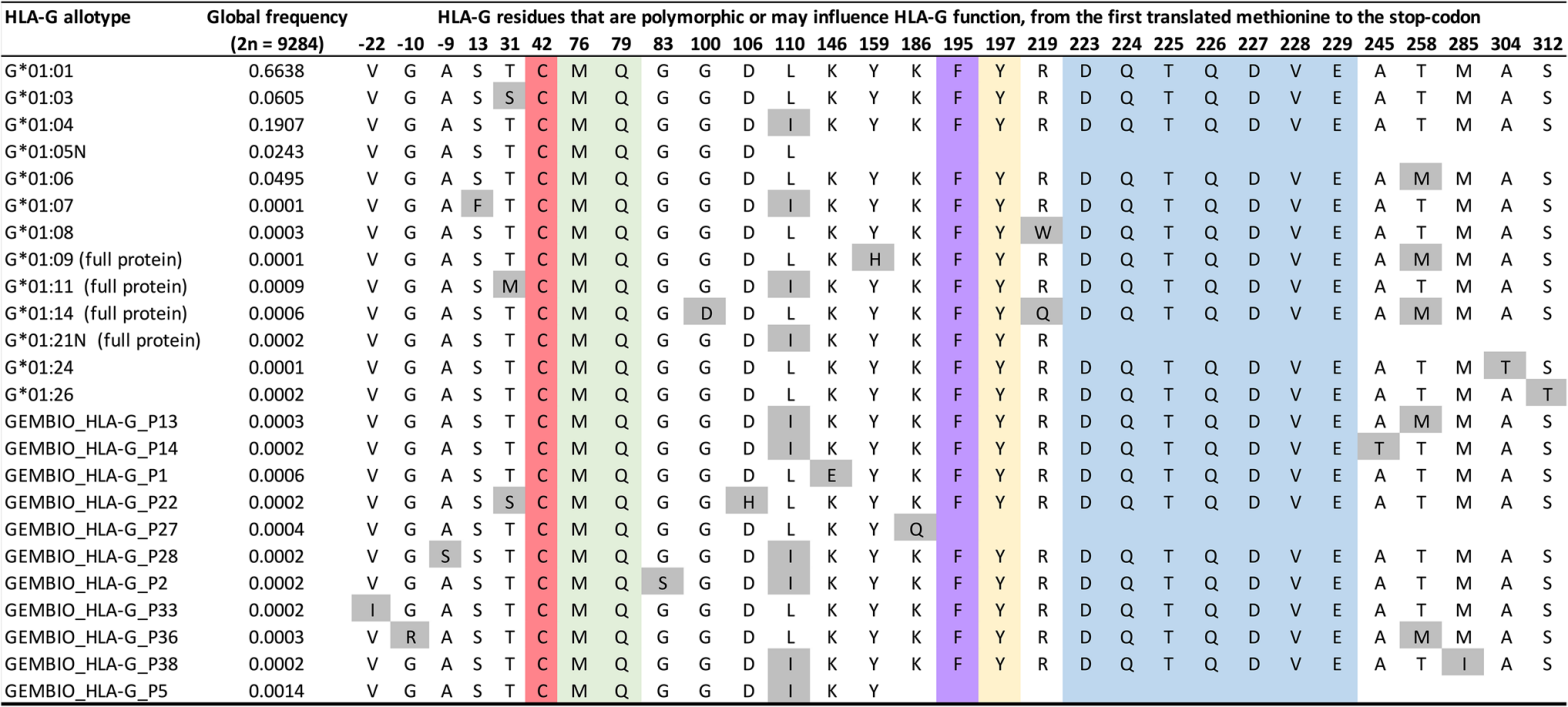
HLA-G residues that are polymorphic or may influence HLA-G function, considering known allotypes detected in 4642 samples from across the world and new allotypes that have occurred at least twice. Polymorphic residues are marked in shades of gray. Important residues for the HLA-G function are marked in other colors. For allotypes with premature stop-codons, there is no amino acid indication after the frameshift mutation. In red, the Cysteine responsible for dimer formation. In green, the Methionine and Glutamine that interact with KIR2DL4. In purple and yellow, the important residues for ILT-2 and ILT-4 interaction. In blue, the motif DQTQDVE, which interacts with the TCD8 receptor.

**Figure 7:**
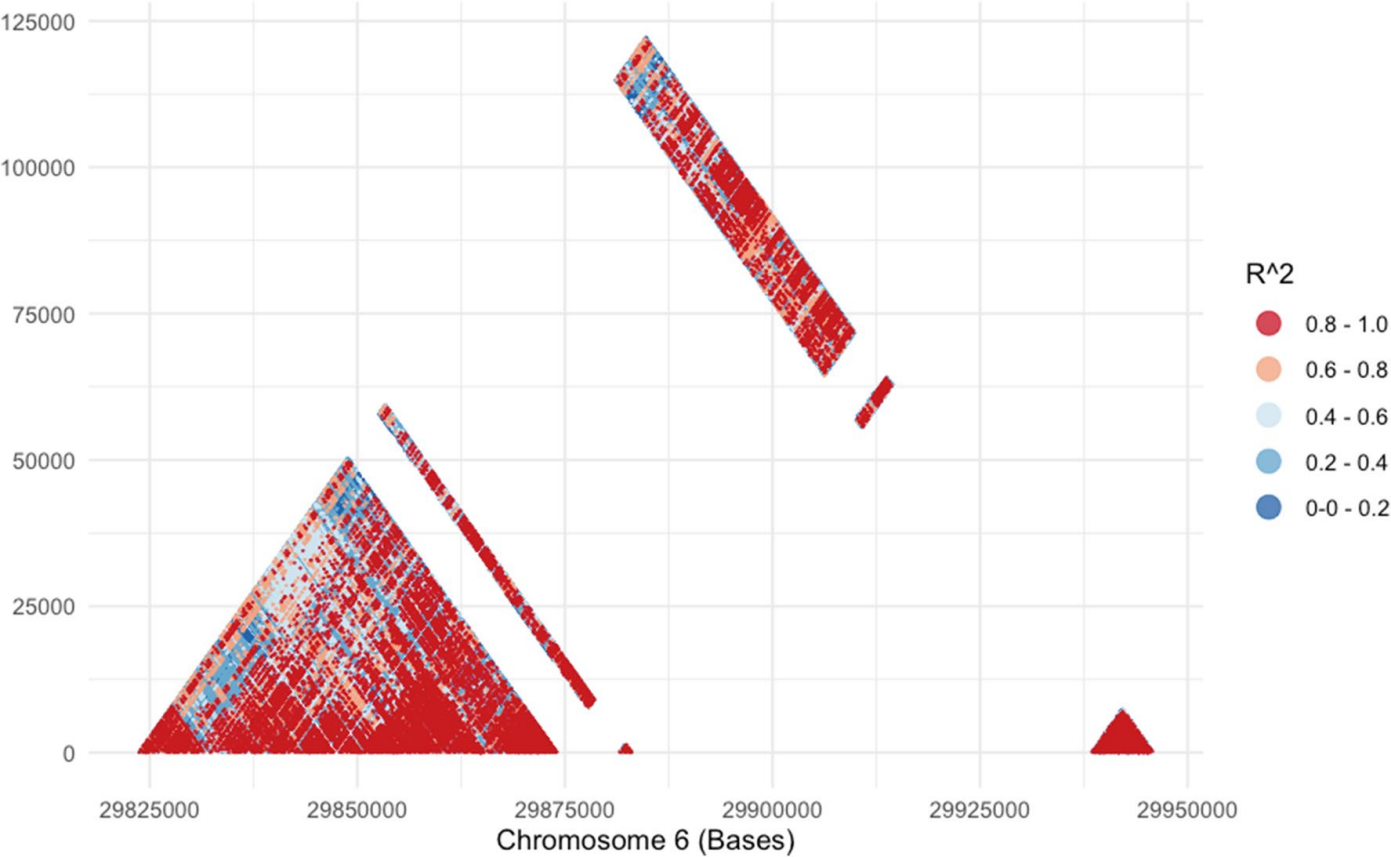
Linkage disequilibrium encompassing the *HLA-G* and *HLA-A* region, considering 5347 individuals from worldwide population samples and SNPs with minimum allele frequency higher than 1%. We have removed variants that coincides with known structural variants, producing three continuous segments: chr6:29,823,675–29,874,064, starting from the *HLA-G* promoter up to 43 Kb downstream *HLA-G*, chr6:29,881,527–29,883,079, a 1.5 kb region between *HLA-A* and *HLA-G*, and chr6:29,938,412–29,945,862, starting 4 kb upstream *HLA-A* to the end of the *HLA-A* 3’UTR.

In Fig. 6, we highlight all the important amino acid residues that may influence HLA-G function, and the polymorphic residues for allotypes that occurred at least twice in our dataset. Among these critical residues for HLA-G function, we may cite the Cysteine at the α1 domain (position 42 in the mature protein), which is responsible for dimer formation (C42-C42) among HLA-G membrane-bound and soluble isoforms, and dimers interact with more affinity with HLA-G receptors^4^. The Methionine and Glutamine (76 and 79 at the mature protein, in green in Fig. 6) residues, also at the α1 domain, interact with KIR2DL4^52^. The residues 195 and 197, at the α3 domain, interact with ILT-2 and ILT-4 leukocyte receptors (purple and yellow in Fig. 7, respectively)^53^. The motif DQTQDVE, also at the α3 domain, interacts with the TCD8 receptor (in blue in Fig. 6)^54^. As can be observed, there is no variability at residues associated with HLA-G major biological properties in a worldwide sample. Note that truncated proteins lacking the α3 domain (G*01:05 N, G*01:21 N, and some new allotypes) present limited interaction with HLA-G receptors. However, the global frequency of these truncated allotypes is low (2.6%), mostly represented by G*01:05 N.

### The *HLA-G* 3’ untranslated region

The 3’ untranslated region (3’UTR) of most of the mRNA transcripts is the primary target for microRNAs (miRNA), which are small non-coding RNA molecules that post-transcriptionally modulate gene expression levels. MiRNAs modulate gene expression by two different mechanisms, mRNA degradation and translation blockage^55^. Variants in both miRNA coding gene and miRNA target sequence may influence miRNA/mRNA binding strength and thus influence gene expression levels^56^.

The *HLA-G* 3’UTR segment was fully characterized in 2010 by Castelli and colleagues, analyzing a large admixed Brazilian sample using Sanger sequencing^41^. There were eight frequent haplotypes in that survey, named UTR-01 to UTR-08, in order of frequency. Each of these 3’UTR haplotypes was in LD with specific *HLA-G* coding region alleles. The existence of these frequent haplotypes and their associated alleles were further confirmed by many other studies, using Sanger sequencing and NGS in different populations^24,27,28,30,40,57–67^. There is a high LD between haplotypes from the *HLA-G* 3’UTR and the coding region (Fig. 1, Table 4), with each 3’UTR haplotype following specific *HLA-G* coding alleles, such as G*01:01:01:01-UTR-01, G*01:01:01:04-UTR-06, G*01:01:01:05-UTR-04, G*01:03-UTR-05, G*01:04-UTR-03, and G*01:01:03-UTR-07^41^.

**Table 4:** The relationship between *HLA-G* alleles and 3’UTR haplotypes, for combinations that have occurred at least twice in 4640 individuals across the globe.

*HLA-G* allele	3'UTR haplotype	Global frequency^a^	Internal frequency^b^
G*01:01:01:01	UTR-06	0.0027	0.0116
G*01:01:01:01	UTR-60	0.0002	0.0009
G*01:01:01:01	UTR-01	0.2277	0.9846
G*01:01:01:04	UTR-20	0.0036	0.1000
G*01:01:01:04	UTR-18	0.0198	0.5576
G*01:01:01:04	UTR-06	0.0121	0.3394
G*01:01:01:05	UTR-04	0.0704	0.9985
G*01:01:01:06	UTR-27	0.0002	0.0161
G*01:01:01:06	UTR-04	0.0131	0.9839
G*01:01:01:08	UTR-01	0.0204	1.0000
G*01:01:01:09	UTR-01	0.0033	1.0000
G*01:01:01:13	UTR-06	0.0066	1.0000
G*01:01:01:14Q	UTR-01	0.0004	1.0000
G*01:01:02:01	UTR-02	0.1470	0.9956
G*01:01:02:01	UTR-10	0.0002	0.0015
G*01:01:02:02	UTR-02	0.0045	1.0000
G*01:01:02:04	UTR-02	0.0015	1.0000
G*01:01:03:03	UTR-07	0.0744	0.9957
G*01:01:03:03	UTR-31	0.0002	0.0029
G*01:01:03:04	UTR-07	0.0002	1.0000
G*01:01:12	UTR-02	0.0022	1.0000
G*01:01:14	UTR-02	0.0025	1.0000
G*01:01:15	UTR-06	0.0039	1.0000
G*01:01:17	UTR-02	0.0026	1.0000
G*01:01:19	UTR-02	0.0017	1.0000
G*01:01:22:01	UTR-02	0.0128	1.0000
G*01:01:22:04	UTR-02	0.0004	1.0000
G*01:03:01:02	UTR-56	0.0029	0.0510
G*01:03:01:02	UTR-17	0.0038	0.0662
G*01:03:01:02	UTR-48	0.0004	0.0076
G*01:03:01:02	UTR-05	0.0499	0.8752
G*01:04:01:01	UTR-03	0.1211	0.9799
G*01:04:01:01	UTR-02	0.0004	0.0035
G*01:04:01:01	UTR-13	0.0018	0.0148
G*01:04:01:02	UTR-53	0.0002	0.0227
G*01:04:01:02	UTR-03	0.0093	0.9773
G*01:04:04	UTR-23	0.0009	0.0171
G*01:04:04	UTR-03	0.0495	0.9829
G*01:04:05	UTR-03	0.0027	1.0000
G*01:05 N	UTR-02	0.0243	1.0000
G*01:06:01:01	UTR-02	0.0484	1.0000
G*01:06:01:02	UTR-02	0.0009	1.0000
G*01:08:02	UTR-02	0.0003	1.0000
G*01:11	UTR-03	0.0009	1.0000
G*01:14	UTR-05	0.0006	1.0000
G*01:21 N	UTR-03	0.0002	1.0000
G*01:26	UTR-02	0.0002	1.0000

^a^The global frequency of this haplotype.

^b^Considering all the 3’UTR haplotypes associated with a given genomic allele, this is the frequency in which this specific 3’UTR haplotype follows the given genomic allele. An internal frequency of 1.000 indicates that the given genomic allele always presents the same 3’UTR sequence.

Although two consecutive exons form the *HLA-G* 3’UTR, the sequence after the stop codon at exon 6 does not present any variant. Thus, for the 3’UTR analysis, we have considered only the last *HLA-G* exon. According to transcripts NM_001363567 and NM_002127, the last *HLA-G* exon extends up to position 6:29,831,122 (hg38), which corresponds to the *HLA-G* position + 3292, when we consider the presence of the 14 bp sequence^15^. Our data reinforce the presence of few nucleotide exchanges in the *HLA-G* 3’UTR, with 27 variants distributed across the last *HLA-G* exon. Of these, only nine are recognized as polymorphisms, with a global frequency superior to 1%. Supplementary Table S2 displays the global frequency of each of these variants.

The 27 variants form 35 different haplotypes, with different frequencies across the globe, as shown in Fig. 4 and Table S5. However, as initially described in 2010, only eight of these haplotypes are globally frequent (Fig. 4), with frequencies varying in different biogeographic regions. UTR-01 and UTR-02 are common in Europe, Africa, America, and parts of Asia, and less common in Southeast Asia and Oceania. UTR-01 and UTR-02 are the most divergent ones, but they are in LD with the HLA-G*01:01:01:01 and G*01:01:02:01 coding alleles (encoding the same G*01:01 allotype), which in turn are also the most divergent coding region alleles. Conversely, the frequencies of UTR-03 and -07 follow the opposite path in terms of worldwide distribution, with the first associated with allotype G*01:04 and the last with G*01:01. UTR-07 is absent in Africa (Fig. 4).

The *HLA-G* 3’UTR presents at least three variants that have been evaluated in terms of functional studies. Undoubtedly, the most studied is the presence or absence of a 14-bp fragment at the beginning of the last *HLA-G* exon, commonly referred to as the 14 bp INS/DEL (rs371194629) polymorphism. The ancestral allele is the presence of this sequence, which is detectable in most primates. The supplementary alignment provides its status in each 3’UTR sequence. The DEL allele is associated with the frequent UTR-01, UTR-03, UTR-04, UTR-06, and UTR-18 haplotypes (Fig. 4), among others. The ancestral allele (INS, presence) was associated with lower HLA-G production of the membrane-bound and soluble isoforms^68–71^, while other studies pointed the opposite^72,73^. Previous studies indicated that the 14-base sequence (or another variant in linkage with it) triggers alternative splicing removing from the mature *HLA-G* mRNA the first 92 bases of the last exon, which also includes the 14b sequence^74^. These alternative mRNA seems to be more stable^75^, but the fraction of mRNA that undergoes this alternative splicing varies in different cell lines and tissues. Moreover, the 14-bp polymorphism may influence the binding of a few miRNAs that target the 14b sequence directly, in addition to various miRNAs that target the 92b segment that may be spliced out when the 14b sequence is present^76,77^. However, with the exception of miR-133a^78^, none of these interactions have been proven functionally.

The + *3142 G* > *C* variant (rs1063320) influences the binding of some miRNAs, with Guanine allele favoring the targeting of three miRNAs, miR-148a-3p, miR-148b-3p, and miR-152^56,76^. However, while studies confirm that these miRNAs down-regulate HLA-G expression^79,80^, apparently, they down-regulate HLA-G irrespectively of the nucleotide at position + 3142^80^. Interestingly, miR-148a-3p and miR-148b also influence *HLA-C* expression, and both genes are co-expressed in the placenta during pregnancy^81^. The allele associated with lower miRNA binding, + 3142C, can be found worldwide and co-occurs with the frequent UTR-01, UTR-04, UTR-06, and UTR-18 haplotypes (Fig. 4, supplementary alignment).

The + 3187 A > G variant (rs9380142) coincides with an AU-rich motif in the HLA-G 3’UTR that may trigger *HLA-G* mRNA degradation. The presence of Guanine in this position reduces the size of this AU-rich motif, positively influencing mRNA stability and associated with higher *HLA-G* expression^82^. The only frequent 3’UTR carrying the high-expressing allele (Guanine) is UTR-01.

Many studies addressed the influence of the 3’UTR sequence on the HLA-G expression levels of both membrane-bound and soluble isoforms. Since *HLA-G* presents a high LD (Fig. 1), any study addressing HLA-G expression profile and polymorphism must consider how these polymorphisms interact with each other as haplotypes. For instance, while UTR-04 and UTR-06 present the high-expressing + 3142 > C allele, they also carry the low-expressing + 3187 > A allele. The 14 bp INS allele usually co-occurs with the low expressing + 3142 > G and + 3187 > A alleles, with rare exceptions. The most divergent haplotypes, UTR-01 and UTR-02, differ in all the variants functionally reported to influence HLA-G expression and in two other positions (supplementary alignment). The coding alleles associated with these haplotypes encode the same HLA-G*01:01 allotype. It seems that the frequent 3’UTR haplotypes detected in modern humans somehow present polymorphisms that tune *HLA-G* expression in a dependent manner, possibly resulting in either high or low expression levels depending on the stimuli and the physiological microenvironment. The influence of specific haplotypes on the HLA-G expression levels has already been demonstrated functionally^24^.

The relationship between the *HLA-G* 3’UTR haplotypes and *HLA-G* alleles was addressed in many studies^24,27,28,30,40–42,57–67^, and was confirmed and updated in this survey (Table 4). Some *HLA-G* alleles, such as G*01:01:01:08, G*01:01:22:01, G*01:05 N, and G*01:06:01:01, always carry the same 3’UTR haplotype, while others, such as G*01:01:01:01, G*01:01:01:05, and G*01:01:01:06, are mostly, but not exclusively, associated with a single 3’UTR haplotype. Conversely, the G*01:01:01:04 and G*01:03:01:02 alleles present more than one frequent 3’UTR haplotype. Because of the impact of the 3’UTR sequence on the HLA-G function, this region should be included in the IPD-IMGT/HLA database, and the names of alleles carrying more than one 3’UTR haplotype should be updated. Table 4 lists only the haplotypes with a global frequency higher than 0.5%, which achieved a summed frequency of 95.3%. Following the internal frequencies presented in Table 4, one may argue that it is possible to input the HLA-G allotypes by observing a few SNPs at the 3’UTR segment. Table S5 presents a complete list of all genomic HLA-G alleles and their 3’UTR sequences.

### Linkage disequilibrium between *HLA-G* and *HLA-A*

*HLA-A* is a classical class I gene that encodes a key molecule for antigen presentation. *HLA-A* is the second most variable histocompatibility gene, with more than 6000 alleles already described in the IPD-IMGT/HLA Database. As antigen presentation is the main function of *HLA-A*, it is common sense that natural selection has shaped the genetic variability in its coding region, increasing variability at the peptide binding site, thus allowing a great diversity of antigen presentation^83,84^.

*HLA-A* and *HLA-G* are only 100 Kb apart from each other. This proximity results in high LD and hitchhiking effects might even involve common regulatory regions. Linkage disequilibrium between *HLA-G* and *HLA-A* has been previously demonstrated using low *HLA-A* typing resolution^43^. Here, we demonstrate the LD level between these two genes by using forefront technology. First, we processed the region encompassing *HLA-G* and *HLA-A* using the same pipeline, as described in the supplementary methods. Then, we removed variants laying on regions with known structural variants and rare variants (MAF < 1%), resulting in 1873 frequent SNPs covering the *HLA-G*, *HLA-A*, and their intergenic region. With this data, we calculated the *r*^*2*^ between pairs of bi-allelic SNPs (Fig. 7). Because we removed SNPs in regions with structural variants, we have 3 blocks of continuous variants: (a) chr6:29,823,675–29,874,064, starting from the *HLA-G* promoter up to 43 Kb downstream *HLA-G*, (b) chr6:29,881,527–29,883,079, a 1.5 kb region between *HLA-A* and *HLA-G*, and (c) chr6:29,938,412–29,945,862, starting 4 kb upstream *HLA-A* to the end of the *HLA-A* 3’UTR. Figure 7 demonstrates that the high LD across *HLA-G* extends from its promoter region up to *HLA-A*, with most pairwise *r*^*2*^ higher than 0.6.

This analysis also revealed some new insights involving the relationship between *HLA-G* alleles and HLA-A allotypes. For instance, there is an absolute LD (or almost absolute) between G*01:01:01:05 and A*03 (particularly A*03:01), G*01:01:01:08 and A*30:02, G*01:01:01:13 and A*29:01, G*01:01:03:03 and A*11 (particularly A*11:01), G*01:01:02:01 and A*32:01, G*01:05 N and A*30:01, and G*01:06:01:01 and A*01:01. Conversely, the most frequent *HLA-G* alleles G*01:01:01:01, G*01:01:02:01, G*01:03:01:02, and G*01:04:01:01 are associated with multiple non-overlapping HLA-A allotypes. Therefore, it becomes clear that the LD between *HLA-G* and *HLA-A* is stronger than originally perceived*.*

Balancing selection has been well documented for both *HLA-A*^83,85^ and *HLA-G*^86^*,* but at different fashions. While the most polymorphic segment at *HLA-A* is the coding sequence, associated with the presence of frequent and divergent coding alleles in worldwide populations, the most polymorphic sites at *HLA-G* are the regulatory regions (i.e., promoter, introns, and the 3’UTR, Fig. 2). Many studies have detected signatures of balancing selection at the *HLA-G* promoter and 3’UTR^40,66,86,87^, while the coding segment is conserved, as we demonstrated here, being under purifying selection. However, it is not clear whether balancing selection is indeed operating at the *HLA-G* promoter (for instance, in the region encompassing polymorphism − 725), or whether these findings are due to a hitchhiking effect caused by the selective pressures acting the neighboring *HLA-A* gene.

Since HLA-A is a crucial molecule for triggering adaptive immune responses, *HLA-A* is usually a target for natural selection. Balancing selection is an important force that maintains advantageous genetic diversity in populations, including variations responsible for long-term adaptation to the environment^88^. For the coding region, it is well established that balancing selection deals with an excess of non-synonymous substitutions and an increase of functionally relevant variability.

A previous study from our group has proposed that the *HLA-A locus* is not influencing *HLA-G*, and therefore *HLA-G* is a direct target of selection^87^. However, the study above addressed this issue using the 1000Genomes phase 1 data, which was the only dataset available at the time. Since then, several studies have revealed that the 1000genomes phase I dataset at the HLA region is strongly affected by mapping bias. This mapping bias, together with the fact that those samples were sequenced in a low coverage fashion, and no specific tools for HLA were applied, might have led to a wrong conclusion that *HLA-A* does not influence *HLA-G*.

Here, we demonstrate a strong LD between *HLA-G* and *HLA-A* in a way that the most frequent *HLA-G* alleles are associated with specific *HLA-A* alleles. Because there is a strong LD between any *HLA-G* allele and specific promoter and 3’UTR sequences, balancing selection operating at the *HLA-A locus* may also shapes the *HLA-G* promoter frequencies, downplaying its direct role on *HLA-G*. The same rationale can be applied to the *HLA-G* intronic sequences. High heterozygosity in *HLA-A*, which is commonly observed in worldwide populations, would lead to high heterozygosity in the *HLA-G* coding region (at the DNA level) and, thus, to high heterozygosity in the *HLA-G* promoter and intronic regions^30,40^. We cannot disregard the possibility that part of the genetic signature of balancing selection on *HLA-A* is due to *HLA-G* since the signatures of Natural Selection observed in both genes are not independent, as previous stated.

### Implication of the *HLA-G* variability in worldwide populations

After fully sequencing *HLA-G* and its promoter region in 4640 individuals from 88 different populations samples, we have concluded that this gene is highly conserved in the protein level, with no variation in the residues responsible for the biological function of the protein, i.e., dimer formation, alternative splicing, peptide coupling, and interaction with HLA-G receptors. Considering that HLA-G dimer interaction with HLA-G receptors is more stable than with monomers^17,53^, and considering that dimer formation primarily depends on the disulfide bridge formed between Cysteine residues (C42–C42)^53^, as seen in Fig. 6, all complete proteins detected in worldwide samples exhibit Cysteine at residue 42. Then, theoretically, all proteins and the most common HLA-G isoforms exhibiting the α1 domain may form dimers. Since the mechanisms of *HLA-G* alternative splicing have not been completely defined, the frequent variants in the *HLA-G* introns may influence splicing, yielding different isoforms with different properties. In this context, some of these isoforms may not present the α1 domain^17^, avoiding dimer formation. Most of the *HLA-G* coding region variability coincides with introns (Fig. 2 and Table 2), and it is possible that these variants influence *HLA-G* regulation. All proteins we have detected exhibit no variability at residue motifs described to interact with ILT-2 (Phe195), ILT-4 (Phe195, Tyr197), KIR2DL4 (Met76, Gln79) and CD8 (DQTQDVE, residues 223–229)^2,54,89^. Nevertheless, isoforms presenting an incomplete α1 domain cannot interact with KIR2DL4, particularly the truncated HLA-G proteins encoded by the HLA-G*01:05 N, *01:13 N, and 01:21 N alleles (Fig. 6), as well as with ILTs and CD8 receptors.

Because HLA-G is a promiscuous immune checkpoint molecule, the modulation of its function may be tuned according to specific therapeutical approaches. The use of monoclonal antibodies against HLA-G in situations in which its expression is undesirable (for instance, in cancers and chronic viral disorders) would have many side effects since HLA-G is constitutively expressed in thymus, pancreas, and hematopoietic stem cells^17^. On the other hand, the use of recombinant HLA-G would be a plausible alternative for allograft and autoimmune disease treatment. Since few coding allotypes (proteins) are most frequently observed in worldwide populations, the production of a small number of recombinant HLA-G proteins would be sufficient to individualize treatment, according to the patient *HLA-G* coding region typing. Besides targeting the molecule, it is also possible to modulate its expression taking advantage of the knowledge of the target sites for transcription and post-transcription factors that act on the *HLA-G* gene expression. Considering the canonical and non-canonical miRNA and transcription factor^22,24,56,76,90–93^ targeting *HLA-G*, a comprehensive survey of the *HLA-G* genetic diversity as provided in this study may help to unveil regulatory factors and strategies to differentially modulate HLA-G production whenever necessary.

Taken together, the results of this investigation indicate that: (1) most of the *HLA-G* polymorphic sites are located in the regulatory and intronic regions, (2) although some promoter segments present the highest nucleotide diversity and Tajima’s *D*, the strongest signals of balancing selection come from intronic segments, particularly intron 5 (3) a high LD is observed across *HLA-G*, from 4 Kb upstream the gene up to the *HLA-A* locus, (4) it is possible that balancing selection acting on *HLA-A* may be influencing the high heterozygosity observed in the regulatory and intronic sequences of *HLA-G*, and vice-versa, (5) the presence of few proteins frequently observed in worldwide populations corroborates the role of the HLA-G as an immune checkpoint molecule rather than as an antigen-presenting molecule; (6) the lack of variation in residues responsible for major HLA-G biological functions reinforces the immunomodulatory properties of the molecule, maintained throughout evolution; (7) most common variation sites within *HLA-G* regulatory and coding region have been maintained as haplotypes, as observed in worldwide populations, (8) motifs associated with HLA-G interaction with receptors may be engineered modified to impede or enhance receptor interaction according to the underlying disorder, (9) possible therapeutic use of HLA-G as an immune checkpoint agent may be accomplished with the production of a limited number of recombinant proteins.

## Concluding remarks

This is the most comprehensive study of the genetic diversity involving *HLA-G* performed so far. This large dataset, composed of populations from different ancestries and biogeographical regions and using sequencing data produced with cutting-edge sequencing technology and bioinformatics pipelines, may provide a broad picture of worldwide patterns of *HLA-G* haplotype distribution and linkage disequilibrium. This landmark achievement on *HLA-G* genetic diversity that will hardly be overridden in the near future provides all the basis for in-depth association and functional studies, which is of utmost importance given the immunomodulatory properties of the molecule.

## Supplementary Information

Supplementary Information.

Supplementary Table S2.

Supplementary Table S3.

Supplementary Table S4.

Supplementary Table S5.
