# Supplementary material for: HLA-G genetic diversity and evolutive aspects in worldwide populations
Source: Sci Rep. 2021 Nov 29;11:23070. doi: 10.1038/s41598-021-02106-4 (PMC8629979; doi:10.1038/s41598-021-02106-4)
Supplement: Supplementary file 1 — Supplementary Information. [file 41598_2021_2106_MOESM1_ESM.docx]

*HLA-G* genetic diversity and evolutive aspects in worldwide populations

Erick C. Castelli ^1,2 *^, Bibiana S. de Almeida ^3, 4^, Yara C. N. Muniz ^5^, Nayane S. B. Silva ^1^, Marília R. S. Passos ^1^, Andreia S. Souza ^1^, Abigail E. Page ^6^, Mark Dyble ^7^, Daniel Smith ^8^, Gabriela Aguileta ^9^, Jaume Bertranpetit ^9^, Andrea B. Migliano ^10^, Yeda A. O. Duarte ^11^, Marília O. Scliar ^12^, Jaqueline Wang ^12^, Maria Rita Passos-Bueno ^12,13^, Michel S. Naslavsky ^12,13,14^, Mayana Zatz ^12,13^, Celso Teixeira Mendes-Junior ^15^, Eduardo A. Donadi ^3 *^

^1^ São Paulo State University (UNESP), Molecular Genetics and Bioinformatics Laboratory, Experimental Research Unit, School of Medicine, Botucatu, State of São Paulo, Brazil

^2^ São Paulo State University (UNESP), Department of Pathology, School of Medicine, Botucatu, Brazil

^3^ Division of Clinical Immunology, Department of Medicine, Ribeirão Preto Medical School, University of São Paulo (USP), 14049-900 Ribeirão Preto, SP, Brazil.

^4^ Laboratório Multiusuário de Estudos em Biologia, Centro de Ciências Biológicas, Universidade Federal de Santa Catarina (UFSC), Florianópolis, Brazil.

^5^ Departamento de Biologia Celular, Embriologia e Genética, Centro de Ciências Biológicas, Universidade Federal de Santa Catarina (UFSC), Florianópolis, Brazil.

^6^ Mark

^7^ Departament of Anthropology, University College London (UCL), London, UK

^8^ Bristol Medical School (PHS), University of Bristol, Bristol, UK

^9^ Department of Experimental and Health Sciences, Universitat Pompeu Fabra, Barcelona

^10^ Departament of Anthropology, Unversity of Zurich, Zurich, Switzerland

^11^ Escola de Enfermagem e Faculdade de Saúde Pública, Universidade de São Paulo (USP), São Paulo, Brazil

^12^ Human Genome and Stem Cell Research Center, Biosciences Institute, University of São Paulo (USP), São Paulo, State of São Paulo, Brazil

^13^ Department of Genetics and Evolutionary Biology, Biosciences Institute, University of São Paulo (USP), São Paulo, Brazil

^14^ Hospital Israelita Albert Einstein, São Paulo, State of São Paulo, Brazil

^15^ Faculdade de Filosofia Ciências e Letras de Ribeirão Preto (FFCLRP), Universidade de São Paulo (USP), 14049-900, Ribeirão Preto, SP, Brazil.

* Correspondence: Erick C. Castelli (erick.castelli@unesp.br) and Eduardo A. Donadi (eadonadi@fmrp.usp.br)

[**Supplementary methods** 2](#_Toc84423539)

[Data download from 1000Genomes and HGDP 2](#_Toc84423540)

[Sequencing data pre-processing 2](#_Toc84423541)

[Alignment optimization for HLA genes 2](#_Toc84423542)

[Variant call and refinement 3](#_Toc84423543)

[Haplotype calls 3](#_Toc84423544)

[HLA allele definition 4](#_Toc84423545)

[Linkage Disequilibrium 4](#_Toc84423546)

[Nucleotide diversity, number of segregation sites, and Tajima’s D 5](#_Toc84423547)

[Multidimensional Scaling (MDS) 5](#_Toc84423548)

[**Supplementary results** 6](#_Toc84423549)

[Figure S1 6](#_Toc84423550)

[Table S1: The list of population samples evaluated for *HLA-G* genetic diversity and their sample sizes. 6](#_Toc84423551)

[Table S2: List of HLA-G variable sites on 4640 individuals from 88 worldwide population samples, in EXCEL format. 8](#_Toc84423552)

[Table S3: The frequency of HLA-G coding alleles (4-field) in each population sample, country, and biogeographic region, in EXCEL format. 8](#_Toc84423553)

[Table S4: The frequency of HLA-G CDS allotype (2-field) in each population sample, country, and biogeographic region, in EXCEL format. 8](#_Toc84423554)

[Table S5: The frequency of HLA-G 3’UTR haplotypes in each population sample, country, and biogeographic region, in EXCEL format. 8](#_Toc84423555)

[**References** 8](#_Toc84423556)

# **Supplementary methods**

## Data download from 1000Genomes and HGDP

We downloaded high depth-of-coverage sequencing data (BAM format) from 831 individuals from the Human Genome Diversity Project [1], by using the links available on <https://www.internationalgenome.org/data-portal/data-collection/hgdp/> and a script that parallelizes download based on *wget* or *aria2*. We also download high depth-of-coverage sequencing data (BAM format) from 2,504 samples from the 1000 Genomes project, using the high-speed protocol ASPERA [2].

## Sequencing data pre-processing

There is a tutorial for calling HLA variants and haplotypes in https://github.com/erickcastelli/HLA_genotyping.

For samples from the SABE cohort [3] and from our dataset of samples from Southeast Asia, we aligned all reads using BWA MEM to the same reference genome used by the 1000 Genomes initiative. For the samples from 1000Genomes and HGDP, we already had a BAM file with the mapping coordinates for the reference genome hg38. In all cases, we used *samtools view* [4] to extract all reads from chromosome 6 and from HLA genes that are included in the reference genome, and also unmapped reads, generating a smaller BAM file for each sample. Each cohort used a different sequencing strategy but generating raw data in the same format, which was post-processed using the same pipeline as described further.

All the procedures we have applied to analyze *HLA-G* and *HLA-A* were performed in Dell workstation, with a 10-core processor and 64 GB of RAM, running Ubuntu Linux 18.04.

## Alignment optimization for HLA genes

We used hla-mapper version 4.0.11 to optimize alignments in the MHC region [5]. Hla-mapper is available for download at [www.castelli-lab.net/apps/hla-mapper](http://www.castelli-lab.net/apps/hla-mapper). The MHC region is prone to alignment and genotyping bias. The input for hla-mapper was the smaller BAM file generated in the previous steps, with the default configuration, and the end product of such optimization is a BAM file for each sample. This procedure minimizes the alignment errors, allowing a more accurate genotyping and haplotyping procedure as described further.

## Variant call and refinement

For variant calling, we used the Genome Analysis Toolkit (GATK), version 4.1.9.0, algorithm HaplotypeCaller, in the GVCF mode and by using default parameters [6]. Then, we joined all G.VCF files into a single G.VCF (algorithm CombinedGVCFs), and converted this G.VCF file to VCF by using GenotypeGVCFs. Then, recoded the VCF file using vcftools to correct minor encoding errors[7]. After, we proceeded with a variant refinement step by first using *vcfx* ([www.castelli-lab.net/apps/vcfx](http://www.castelli-lab.net/apps/vcfx)) algorithm *checkpl* (genotype likelihood = 0.95), which introduces missing alleles in genotypes with low likelihood. Among these we may cite, for instance, homozygous genotype in a region with only two reads, or a heterozygous genotype in which 90% of all reads point to the same nucleotide. After, we used a second *vcfx* algorithm called *evidence* with default parameters, which annotates each variant with quality control parameters such as the number of heterozygous sites with even distribution of reads per allele, the number of homozygous genotypes for alternative alleles, and others. Then, we used *vcfx filter* to recover only variants annotated as PASS or WARN in the previous steps, manually evaluating variants annotated as WARN. The final step for variant refinent is the removal of alternative alleles that are no longer present in the dataset, recoding the VCF file using *bamtools view --trim-alt-alleles --min-ac 1* [8].

## Haplotype calls

To call haplotypes, we combined read-aware phasing and probabilistic models. First, we normalized the VCF file converting multi-allelic variants into bi-allelic ones using *bamtools norm*. Then, we used *GATK ReadBackedPhasing* (from GATK 3.8) to infer the physical phasing between closely related variants. This step can be parallelized using a Perl script to speed up the process, because *ReadBackedPhasing* is not multithreaded. This method is described in details in https://github.com/erickcastelli/phasex. The input for this step is the refined VCF file from the previous step. The VCF file containing the phased data generated by *ReadBackedPhasing* is the input for the haplotyping procedure using a local program named *phasex*.

Phasex is a program that automates multiple haplotyping runs by using Shapeit 4 [9] and the data obtained by *ReadBackedPhasing* or *WhatsHap*. It performs many independent haplotyping runs, comparing the results afterwards, fixing haplotypes for a sample in which at least 95% of the runs indicate the same results. Then, this step is repeated many times until the number of samples with the same haplotype at least 95% of the runs do not increase any further. The end product of this procedure is phased bi-allelic VCF, which is then normalized to multi-allelic variants using *bamtools norm*. It should be mentioned that we have evaluated *HLA-G* and *HLA-A* separately by retrieving the variants corresponding to each loci, and running phasex for each gene. Phasex is available at https://github.com/erickcastelli/phasex.

## HLA allele definition

By using a Perl script to automate  the process, we first exported the phased VCF to complete sequences of each gene. To do that, we used *vcfx fasta*, indicating chromosome 6 as a reference and the phased VCF. This produces two sequences per individual, one for each chromosome. Then, we counted the number of different sequences, their global count, and the samples that present these sequences. Second, we exported the phased VCF to complete CDS sequences (only exons). To do that, we used *vcfx transcript*, indicating chromosome 6 as a reference, the phased VCF, and a BED file with coordinates of each exon (starting from the first translated ATG). This procedure also produces two CDS sequences per individual, one for each chromosome. We also counted the number of different sequences, their global count, and the samples that present these sequences. Third, we translated the CDS sequence of each chromosome using *emboss transeq*, generating a fasta file with the proteins encoded by each chromosome of each individual. The three datasets (genomic, exonic, and protein sequences) are then compared to the sequences available in the IPD-IMGT/HLA Database [10]. When the sequence we detected can be found in the database, we updated its name accordingly. When it was a new sequence, we named the sequence as a new one.

All these steps are automated by the Perl script (available upon request), which generates fasta files as described above, and a final database indicating the alleles of each individual considering the full genomic sequence, the exonic sequence, and the protein sequence.

## Linkage Disequilibrium

We assessed Linkage Disequilibrium (LD) in the *HLA-G* locus by using Haploview [11], and bi-allelic variants with a minor allele frequency of at least 0.1%. Then, we exported the LD plot into SVG format, further processing it using Inkscape to increase font size and get a better readability.

For the relationship between *HLA-G* and *HLA-A*, we used the same pipeline described above to assess the genetic diversity of the region starting from 4kb upstream *HLA-G* to 100 bp downstream *HLA-A*. We removed rare variants (MAF < 1%) and variants that coincides with known structural variants between these two genes. We also applied the phasing procedure as described above. We obtained pairwise r^2^ using Tomohawk (<https://mklarqvist.github.io/tomahawk/>), plotting the r^2^ values using R based on this tutorial (https://www.biostars.org/p/347796/).

## Nucleotide diversity, number of segregation sites, and Tajima’s D

We assessed nucleotide diversity, the number of segregation sites, and Tajima’s D by using *Variscan* [12]*.* Then, the output was processed using R and plot, which were further processed using Inkscape.

## Multidimensional Scaling (MDS)

We used R to perform multidimensional scaling (MDS) based on the *F_ST_* values among pairs of populations, plotting the results using the ggplot2 package. The *F_ST_* values among pairs of populations were measure by *Arlequin* 3.5 [13] . The .ARP file for *Arlequin* was generated using a local Perl script.

# **Supplementary results**


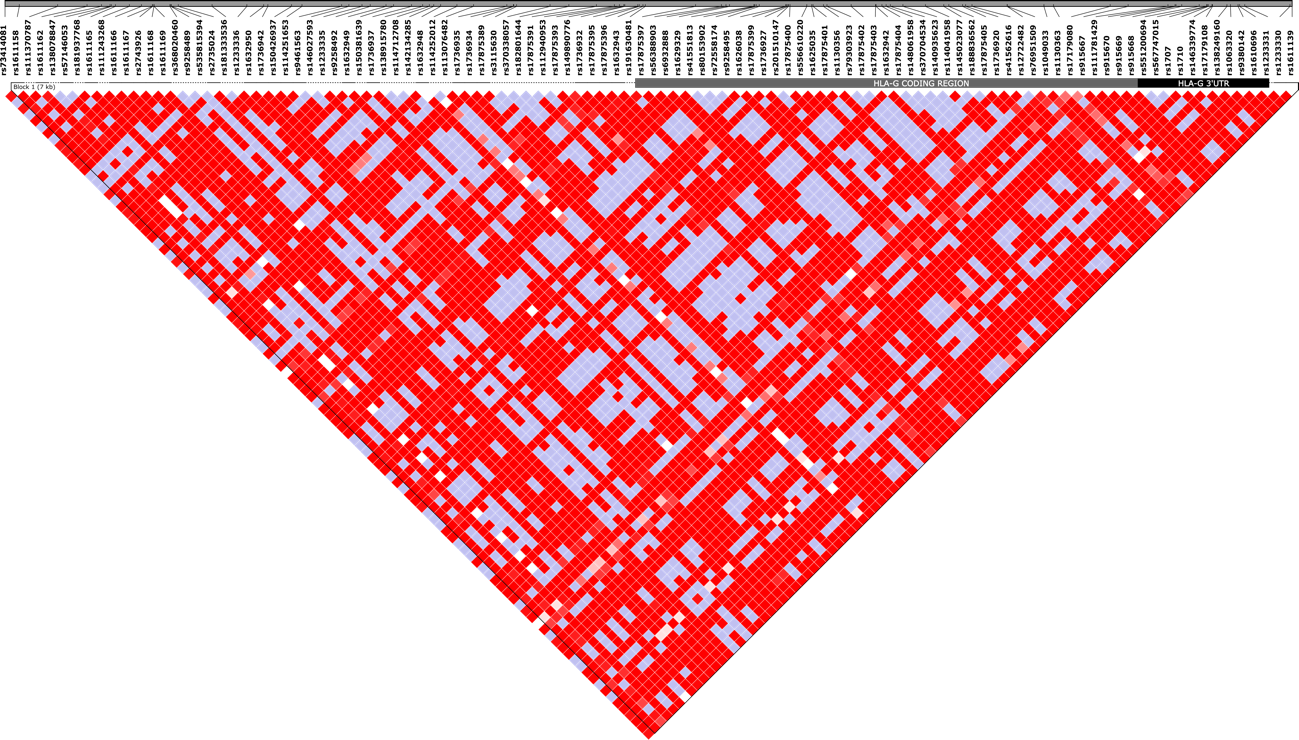


## Figure S1

**Figure S1**: Linkage disequilibrium between pairs of 106 bi-allelic SNPs at the *HLA-G* gene region, starting from 4Kb upstream the gene up to 100 nucleotides downstream, all presenting a global minimum allele frequency of 0.1%. The image was generated in the Haploview and further edited by using Inkscape. Areas in dark red indicate strong LD (LOD > 2, *D’* = 1), shades of pink indicate moderate LD (LOD > 2, *D’* < 1), blue indicates weak LD (LOD < 2, *D’* = 1), and white indicates no LD (LOD < 2, *D’* < 1).

## Table S1: The list of population samples evaluated for *HLA-G* genetic diversity and their sample sizes.

| **Code** | **Population** | **Region** | **Biogeographic Region** | **Resource** | **Size** |
| --- | --- | --- | --- | --- | --- |
| BAA | Bantu in South Africa | South Africa | Africa | IGSR | 4 |
| BAK | Bantu in Kenya | Kenya | Africa | IGSR | 10 |
| BIA | Biaka in Central African Republic | Central African Republic | Africa | IGSR | 24 |
| ESN | Esan | Nigeria | Africa | 1000genomes | 97 |
| GWD | Gambian in Western Divisions in the Gambia | Gambia | Africa | 1000genomes | 112 |
| LWK | Luhya in Webuye, Kenya | Kenya | Africa | 1000genomes | 99 |
| MAN | Mandenka in Senegal | Senegal | Africa | IGSR | 6 |
| MSL | Mende in Sierra Leone | Sierra Leone | Africa | 1000genomes | 85 |
| MTI | Mbuti in Democratic Republic of Congo | Democratic Republic of Congo | Africa | IGSR | 10 |
| SAN | San in Namibia | Namibia | Africa | IGSR | 2 |
| YRI | Yoruba in Ibadan | Nigeria | Africa | 1000genomes | 106 |
| YRN | Yoruba in Nigeria | Nigeria | Africa | IGSR | 17 |
| ACB | African Caribbeans in Barbados | Barbados | America | 1000genomes | 95 |
| ASW | Americans of African Ancestry in SW USA | USA | America | 1000genomes | 61 |
| BRA | Brazilians from São Paulo | Brazil | America | SABE | 1311 |
| CLM | Colombians from Medellin | Colombia | America | 1000genomes | 94 |
| COL | Colombian in Colombia | Colombia | America | IGSR | 5 |
| GIH | Gujarati Indian from Houston, Texas | USA | America | 1000genomes | 103 |
| KAR | Karitiana in Brazil | Brazil | America | IGSR | 9 |
| MAY | Maya in Mexico | Mexico | America | IGSR | 19 |
| MXL | Mexican Ancestry from Los Angeles USA | USA | America | 1000genomes | 64 |
| PEL | Peruvians from Lima, Peru | Peru | America | 1000genomes | 85 |
| PIM | Pima in Mexico | Mexico | America | IGSR | 12 |
| PUR | Puerto Ricans from Puerto Rico | Puerto Rico | America | 1000genomes | 100 |
| SUR | Surui in Brazil | Brazil | America | IGSR | 6 |
| BAL | Balochi in Pakistan | Pakistan | Central South Asia | IGSR | 22 |
| BRH | Brahui in Pakistan | Pakistan | Central South Asia | IGSR | 22 |
| BUR | Burusho in Pakistan | Pakistan | Central South Asia | IGSR | 22 |
| HAZ | Hazara in Pakistan | Pakistan | Central South Asia | IGSR | 17 |
| KAL | Kalash in Pakistan | Pakistan | Central South Asia | IGSR | 21 |
| MKP | Makrani in Pakistan | Pakistan | Central South Asia | IGSR | 23 |
| PAT | Pathan in Pakistan | Pakistan | Central South Asia | IGSR | 20 |
| SIN | Sindhi in Pakistan | Pakistan | Central South Asia | IGSR | 20 |
| UYG | Uygur in China | China | Central South Asia | IGSR | 8 |
| CAM | Cambodian in Cambodia | Cambodia | East Asia | IGSR | 7 |
| CDX | Chinese Dai in Xishuangbanna | China | East Asia | 1000genomes | 93 |
| CHB | Han Chinese in Beijing | China | East Asia | 1000genomes | 103 |
| CHS | Southern Han Chinese | China | East Asia | 1000genomes | 105 |
| DAI | Dai in China | China | East Asia | IGSR | 4 |
| DAU | Daur in China | China | East Asia | IGSR | 9 |
| HAN | Han in China | China | East Asia | IGSR | 29 |
| HEZ | Hezhen in China | China | East Asia | IGSR | 6 |
| JAP | Japanese in Japan | Japan | East Asia | IGSR | 26 |
| JPT | Japanese in Tokyo | Japan | East Asia | 1000genomes | 104 |
| KHV | Kinh in Ho Chi Minh City | Vietnan | East Asia | 1000genomes | 98 |
| LAH | Lahu in China | China | East Asia | IGSR | 6 |
| MIA | Miao in China | China | East Asia | IGSR | 7 |
| MON | Mongolian in China | China | East Asia | IGSR | 8 |
| NAX | Naxi in China | China | East Asia | IGSR | 6 |
| NHA | Northern Han in China | China | East Asia | IGSR | 10 |
| ORO | Oroqen in China | China | East Asia | IGSR | 7 |
| SHE | She in China | China | East Asia | IGSR | 8 |
| TUC | Tu in China | China | East Asia | IGSR | 8 |
| TUJ | Tujia in China | China | East Asia | IGSR | 8 |
| XIB | Xibo in China | China | East Asia | IGSR | 5 |
| YAK | Yakut in Siberia | Siberia | East Asia | IGSR | 22 |
| YIC | Yi in China | China | East Asia | IGSR | 7 |
| ADY | Adygei in Caucasus, Russia | Russia | Europe | IGSR | 14 |
| BAS | Basque in France | France | Europe | IGSR | 22 |
| BER | Bergamo Italian | Italy | Europe | IGSR | 10 |
| CEU | Utah Residents (CEPH) with European Ancestry | USA | Europe | 1000genomes | 98 |
| FIN | Finnish | Finland | Europe | 1000genomes | 99 |
| FRE | French in France | France | Europe | IGSR | 24 |
| GBR | British in England and Scotland | England | Europe | 1000genomes | 91 |
| IBS | Iberian Population in Spain | Spain | Europe | 1000genomes | 106 |
| ORC | Orcadian in Orkney | Orkney | Europe | IGSR | 12 |
| RUS | Russian in Russia | Russia | Europe | IGSR | 23 |
| SAR | Sardinian in Italy | Italy | Europe | IGSR | 21 |
| TSI | Toscani in Italia | Italy | Europe | 1000genomes | 104 |
| TUS | Tuscan in Italy | Italy | Europe | IGSR | 6 |
| BED | Bedouin in Negev, Israel | Israel | Middle East | IGSR | 42 |
| DRU | Druze in Carmel | Israel | Middle East | IGSR | 33 |
| MOZ | Mozabite in Mzab, Algeria | Algeria | Middle East | IGSR | 26 |
| PAL | Palestinian in Central, Israel | Israel | Middle East | IGSR | 43 |
| BOU | Bougainville in Bougainville | Bougainville Island | Oceania | IGSR | 9 |
| KOI | Koinambe, Papua New Guinea | New Guinea | Oceania | Our data | 11 |
| KOS | Kosipe, Papua New Guinea | New Guinea | Oceania | Our data | 9 |
| BEB | Bengali from Bangladesh | Bangladesh | South Asia | 1000genomes | 84 |
| ITU | Indian Telugu | India | South Asia | 1000genomes | 100 |
| PJL | Punjabi from Lahore | Pakistan | South Asia | 1000genomes | 96 |
| STU | Sri Lankan Tamil | Sri Lanka | South Asia | 1000genomes | 100 |
| AET | Aeta, Negritos | Philippines | Southeast Asia | Our data | 15 |
| AGT | Agta, Negritos | Philippines | Southeast Asia | Our data | 11 |
| BAT | Batak, North Sumatra | Sumatra | Southeast Asia | Our data | 10 |
| JEH | Jehai, Malaysia | Malaysia | Southeast Asia | Our data | 8 |
| KIN | Kintaq, Malaysia | Malaysia | Southeast Asia | Our data | 5 |
| MAM | Mamanwa, Negritos | Philippines | Southeast Asia | Our data | 5 |
| TER | Temiar, Malaysia | Malaysia | Southeast Asia | Our data | 6 |

## Table S2: List of HLA-G variable sites on 4640 individuals from 88 worldwide population samples, in EXCEL format.

## Table S3: The frequency of HLA-G coding alleles (4-field) in each population sample, country, and biogeographic region, in EXCEL format.

## Table S4: The frequency of HLA-G CDS allotype (2-field) in each population sample, country, and biogeographic region, in EXCEL format.

## Table S5: The frequency of HLA-G 3’UTR haplotypes in each population sample, country, and biogeographic region, in EXCEL format.

# **References**

[1] L. Clarke, S. Fairley, X. Zheng-Bradley, I. Streeter, E. Perry, E. Lowy, A.M. Tassé, P. Flicek, The international Genome sample resource (IGSR): A worldwide collection of genome variation incorporating the 1000 Genomes Project data, Nucleic Acids Res. 45 (2017) D854–D859. https://doi.org/10.1093/nar/gkw829.

[2] M. Byrska-Bishop, U.S. Evani, X. Zhao, A.O. Basile, H.J. Abel, A.A. Regier, A. Corvelo, W.E. Clarke, R. Musunuri, K. Nagulapalli, S. Fairley, A. Runnels, L. Winterkorn, E. Lowy-Gallego, P. Flicek, S. Germer, H. Brand, I.M. Hall, M.E. Talkowski, G. Narzisi, M.C. Zody, High coverage whole genome sequencing of the expanded 1000 Genomes Project cohort including 602 trios, BioRxiv. (2021) 2021.02.06.430068. http://biorxiv.org/content/early/2021/02/07/2021.02.06.430068.abstract.

[3] M.S. Naslavsky, M.O. Scliar, G.L. Yamamoto, J. Yu Ting Wang, S. Zverinova, T. Karp, K. Nunes, J. Ricardo Magliocco Ceroni, D. Lima de Carvalho, C. Eduardo da Silva Simões, D. Bozoklian, R. Nonaka, N. dos Santos Brito Silva, A. da Silva Souza, H. de Souza Andrade, M. Rodrigues Silva Passos, C. Ferreira Bannwart Castro, C.T. Mendes-Junior, R.L. V, D. Meyer, P.A. F Galante, V. Guryev, E.C. Castelli, Y.A. O Duarte, M. Rita Passos-Bueno, M. Zatz, P. São Paulo, B. Graduate program, G. Muniz, F. Oswaldo Cruz, I. de Pesquisas René Rachou, B. Horizonte, Whole-genome sequencing of 1,171 elderly admixed individuals from the largest Latin American metropolis (São Paulo, Brazil), Wagner CS Magalhães. (20AD).

[4] H. Li, B. Handsaker, A. Wysoker, T. Fennell, J. Ruan, N. Homer, G. Marth, G. Abecasis, R. Durbin, The Sequence Alignment/Map format and SAMtools, Bioinformatics. 25 (2009) 2078–2079. https://doi.org/10.1093/bioinformatics/btp352.

[5] E.C. Castelli, M.A. Paz, A.S. Souza, J. Ramalho, C.T. Mendes-Junior, Hla-mapper: An application to optimize the mapping of HLA sequences produced by massively parallel sequencing procedures, Hum. Immunol. 79 (2018) 678–684. https://doi.org/10.1016/j.humimm.2018.06.010.

[6] G.A. Van der Auwera, M.O. Carneiro, C. Hartl, R. Poplin, G. del Angel, A. Levy-Moonshine, T. Jordan, K. Shakir, D. Roazen, J. Thibault, E. Banks, K. V. Garimella, D. Altshuler, S. Gabriel, M.A. DePristo, From fastQ data to high-confidence variant calls: The genome analysis toolkit best practices pipeline, Curr. Protoc. Bioinforma. (2013). https://doi.org/10.1002/0471250953.bi1110s43.

[7] P. Danecek, A. Auton, G. Abecasis, C.A. Albers, E. Banks, M.A. DePristo, R.E. Handsaker, G. Lunter, G.T. Marth, S.T. Sherry, G. McVean, R. Durbin, The variant call format and VCFtools, Bioinformatics. 27 (2011) 2156–2158. https://doi.org/10.1093/bioinformatics/btr330.

[8] D.W. Barnett, E.K. Garrison, A.R. Quinlan, M.P. Str̈mberg, G.T. Marth, Bamtools: A C++ API and toolkit for analyzing and managing BAM files, Bioinformatics. 27 (2011) 1691–1692. https://doi.org/10.1093/bioinformatics/btr174.

[9] O. Delaneau, J.F. Zagury, M.R. Robinson, J.L. Marchini, E.T. Dermitzakis, Accurate, scalable and integrative haplotype estimation, Nat. Commun. 10 (2019) 24–29. https://doi.org/10.1038/s41467-019-13225-y.

[10] J. Robinson, J.A. Halliwell, J.D. Hayhurst, P. Flicek, P. Parham, S.G.E. Marsh, The IPD and IMGT/HLA database: Allele variant databases, Nucleic Acids Res. 43 (2015) D423–D431. https://doi.org/10.1093/nar/gku1161.

[11] J.C. Barrett, B. Fry, J. Maller, M.J. Daly, Haploview: Analysis and visualization of LD and haplotype maps, Bioinformatics. 21 (2005) 263–265. https://doi.org/10.1093/bioinformatics/bth457.

[12] A.J. Vilella, A. Blanco-Garcia, S. Hutter, J. Rozas, VariScan: Analysis of evolutionary patterns from large-scale DNA sequence polymorphism data, Bioinformatics. 21 (2005) 2791–2793. https://doi.org/10.1093/bioinformatics/bti403.

[13] L. Excoffier, H.E.L. Lischer, Arlequin suite ver 3.5: A new series of programs to perform population genetics analyses under Linux and Windows, Mol. Ecol. Resour. 10 (2010) 564–567. https://doi.org/10.1111/j.1755-0998.2010.02847.x.
